# Metal-Free Tetraphenylethylene and Dibenzo[g,p]chrysene with Thiazolo[5,4‑*d*] Thiazole-Based Donor–Acceptor Conjugated Microporous Polymers for the Efficient Photocatalytic Synthesis of 2‑Substituted Benzimidazole

**DOI:** 10.1021/acspolymersau.6c00023

**Published:** 2026-03-16

**Authors:** Mohamed Gamal Mohamed, Pei-Tzu Wang, Hira Karim, Shiao-Wei Kuo

**Affiliations:** † Department of Materials and Optoelectronic Science, Center for Functional Polymers and Supramolecular Materials, 34874 National Sun Yat-Sen University, Kaohsiung 804, Taiwan; ‡ Chemistry Department, Faculty of Science, 68796 Assiut University, Assiut 71515, Egypt; § Department of Chemistry, School of Natural Sciences (SNS), 66959 National University of Sciences and Technology (NUST), H-12, Islamabad 44000, Pakistan

**Keywords:** tetraphenylethylene, dibenzo[g,p]chrysene, thiazolo[5,4-*d*]thiazole, conjugated microporous polymers, photocatalytic synthesis

## Abstract

The synthesis of benzimidazole is important because its derivatives are widely used in pharmaceuticals and functional materials due to their strong biological activity and ability to interact with biological targets. Herein, two photoactive conjugated microporous polymers (CMPs), TPE-TzTz and TBN-TzTz, featuring donor–acceptor (D–A) architectures, were successfully synthesized via a Schiff-base condensation reaction between dithiooxamide (DITH) and electron-rich aromatic donors—1,1,2,2-tetrakis­[4-formyl-(1,1́-biphenyl)]­ethane (TPE-4Ph-4CHO) and 2,7,10,15-tetra­(4-formylphenyl) dibenzo­[g,p]­chrysene (TBN-4Ph-4CHO)—integrated with a thiazolo­[5,4-*d*]­thiazole (TzTz) acceptor unit. Brunauer–Emmett–Teller (BET) and thermogravimetric analysis (TGA) revealed that TPE-TzTz and TBN-TzTz CMP possess high specific surface areas of 484 and 419 m^2^ g^–1^, with total pore volumes of 1.2 and 0.2 cm^3^ g^–1^, respectively. These two CMPs also exhibited excellent thermal stability with 10% weight-loss temperatures (T_d10_) of 508 °C for TPE-TzTz CMP and 487 °C for TBN-TzTz CMP. Both TzTz CMPs display extended π-conjugation and efficient charge-carrier separation. Under visible-light irradiation, TPE-TzTz and TBN-TzTz CMPs showed outstanding photocatalytic activity in the oxidative cyclization of *o*-phenylenediamine with different derivatives of benzaldehyde to form benzimidazole derivatives. Notably, the TBN-TzTz CMP exhibited superior photocatalytic performance, which can be attributed to its more planar and rigid donor backbone, leading to enhanced π–π stacking and improved charge-carrier mobility. Furthermore, a plausible photocatalytic reaction mechanism was proposed based on experimental catalytic results and the calculated energy band structures of the CMPs. This study presents an effective strategy for designing D–A conjugated microporous polymers with high photocatalytic activity and expands the application of TzTz-based CMPs in photocatalytic organic transformations.

## Graphical abstract

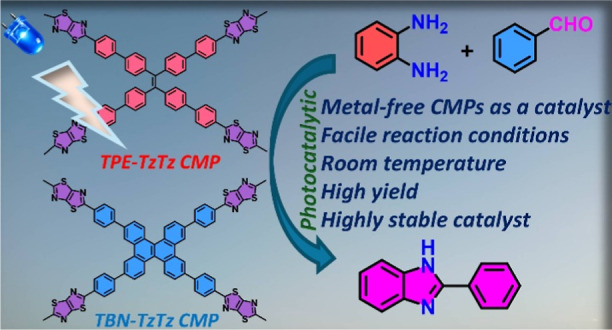

## Introduction

Porous materials constitute an important class of functional materials characterized by a well-developed internal pore structure that has attracted considerable attention in both scientific research and industrial applications. Since the introduction of hyper-cross-linked polymers (HCPs) by Davankov and Tsurupa, a diverse range of organic porous polymers (POPs) has been developed, including CMPs, polymers of intrinsic microporosity (PIMs), covalent organic frameworks (COFs), porous aromatic frameworks (PAFs), and covalent triazine frameworks (CTFs).^1–4^ Among these materials, CMPs are particularly notable due to their extended, rigid π-conjugated backbones that form porous, disordered three-dimensional networks.^5–10^ CMPs are typically composed of lightweight elements and exhibit high specific surface areas, excellent physical and chemical stability, and versatile functionalization potential.^1,2^ Owing to these remarkable properties, CMPs have shown significant promise and progress in multiple applications across different fields, including chemical adsorption and sensing,^11–17^ heterogeneous catalysis,^18–20^ gas adsorption and separation,^21–24^ photoredox catalysis,^25–27^ solar steam generation,^28,29^ energy storage,^30–33^ and biological applications.^34–36^

Benzimidazoles are a class of heterocyclic compounds containing two nitrogen atoms within a fused benzene–imidazole ring system. Hobrecker reported the first synthesis of a benzimidazole, specifically 2,5- and 2,6-dimethylbenzimidazole, marking the discovery of this important *N*-containing heterocycle.^37^ Structurally, benzimidazoles consist of a six-membered benzene ring fused with a five-membered imidazole ring, with two nitrogen atoms located at the 1,3-positions of the fused heterocycle. Notably, their structural similarity to purine derivatives contributes to their wide range of biological activities. Benzimidazole derivatives have been reported to exhibit diverse medicinal properties, including anticancer, antiseptic, antiparasitic, anti-inflammatory, antiviral, antihypertensive, antibacterial, antiulcer, antitumor, anticonvulsant, and enzyme inhibitory activities.^38^ Consequently, numerous pharmaceutical drugs incorporate a benzimidazole moiety into their molecular structure. In recent years, research on benzimidazoles has expanded significantly across multiple disciplines, including biological studies,^39^ dye chemistry,^40^ chemosensory applications,^41^ corrosion science,^42^ and advanced materials science;^43^ owing to their excellent bioavailability, chemical stability, and high efficiency, benzimidazoles have become a crucial topic in contemporary scientific research. Given their broad applicability, considerable efforts have been devoted to developing more efficient and environmentally benign synthetic routes for arylbenzimidazole frameworks.

Among various synthetic approaches, the Weidenhagen reaction has emerged as one of the most promising methods.^44^ This reaction involves the condensation of 1,2-diaminobenzene with aldehydes or ketones in water or organic solvents, typically in the presence of copper acetate or other divalent copper salts as oxidizing agents. Mechanistically, the reaction begins with the formation of an intermediate imine from the aldehyde and 1,2-diaminobenzene, which can equilibrate with a dihydrobenzimidazole species. Subsequently, catalytic oxidative dehydrogenation converts this intermediate to the final benzimidazole product. This method is advantageous due to its relatively mild reaction conditions and operational simplicity. However, a major limitation of many existing synthetic strategies is their dependence on transition metals, particularly precious metals, which increases production costs and hinders large-scale industrial applications. Moreover, the presence of toxic metal residues raises significant concerns in pharmaceutical manufacturing. To address these challenges, researchers are actively developing cost-effective, transition-metal-free, and environmentally sustainable catalytic systems for benzimidazole synthesis.

Thiazolo­[5,4-*d*]­thiazole (TzTz) is a planar, rigid, and electron-deficient fused heteroaromatic unit. Heterocyclic compounds containing nitrogen and sulfur are generally classified as n-type semiconductors with high electron mobility and have therefore attracted considerable attention in the field of organic electronics.^45–47^ Incorporation of the TzTz unit into donor–acceptor (D–A) architectures imparts high oxidative stability and an extended π-conjugated system, which promotes strong intermolecular π–π interactions and facilitates efficient charge transport in solid-state materials.^45–47^ Owing to its electron-deficient nature, TzTz serves as an effective acceptor unit, while its structural rigidity enhances the stability of the resulting materials. These favorable characteristics have been exploited in various applications, particularly in photocatalysis, where TzTz-based frameworks have demonstrated excellent performance in processes such as hydrogen (H_2_) production,^48^ pollutant degradation,^49^ and CO_2_ reduction,^50^ as well as in thin-film electronics.^51^

In this study, TPE-TzTz and TBN-TzTz CMPs, featuring donor–acceptor (D–A) architectures, were successfully synthesized via a Schiff-base condensation reaction [Scheme 1]. Under visible-light irradiation, TPE-TzTz and TBN-TzTz CMPs exhibited outstanding photocatalytic activity in the oxidative cyclization of *o*-phenylenediamine derivatives with different arylaldehydes to form benzimidazole derivatives. We present an innovative synthetic approach for the preparation of benzimidazoles that are both highly efficient and environmentally friendly. This method employs a one-pot synthesis strategy and eliminates the need for any catalysts, thereby simplifying the procedure while reducing its environmental impact.

**Scheme 1:**
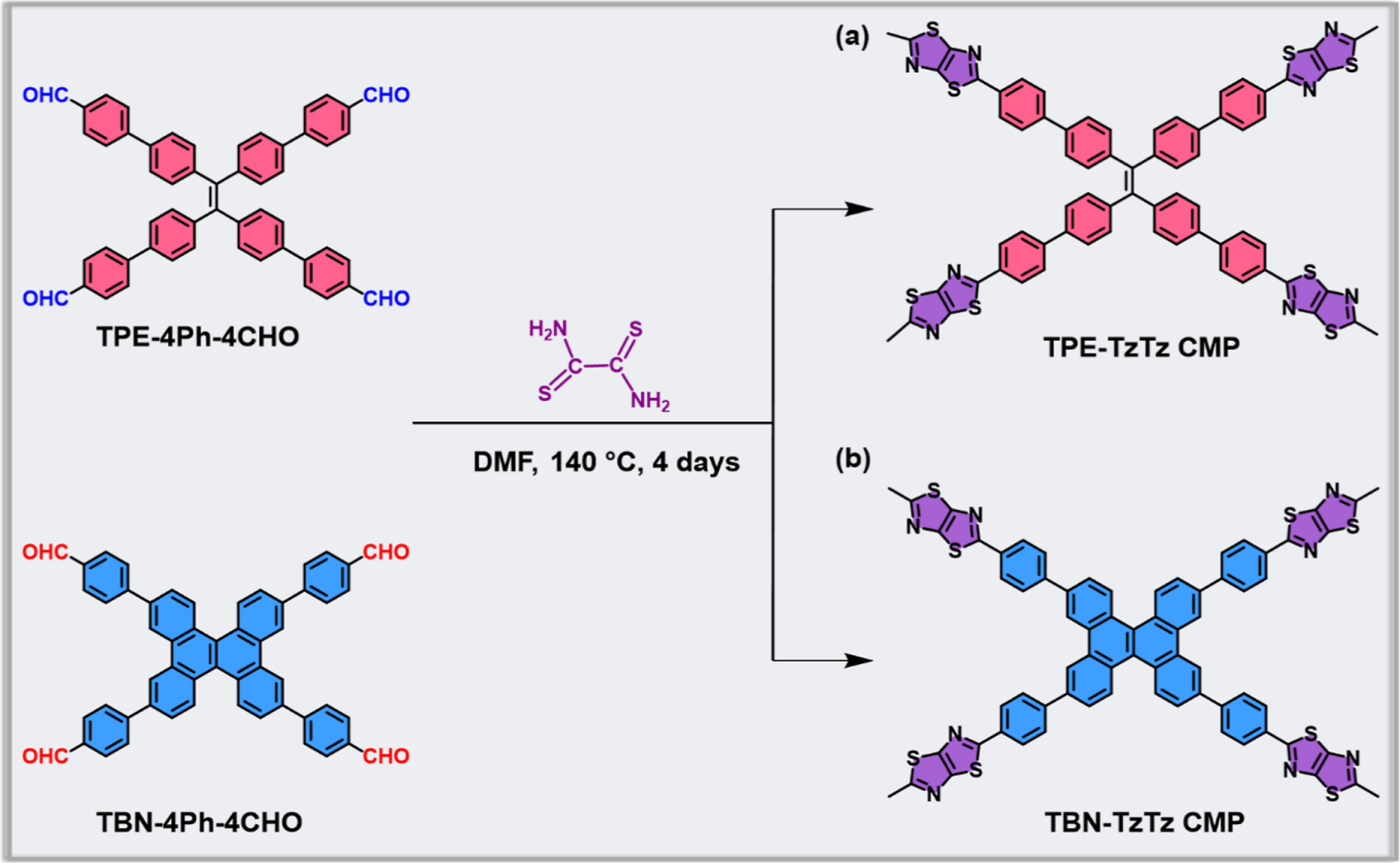
A Preparation Scheme for the Synthesis of (a) TPE-TzTz and (b) TBN-TzTz CMPs Was Developed Using TPE-4Ph-4CHO and TBN-4Ph-4CHO as Precursors, Which Were Polymerized with Dithiooxamide (DITH) through a Condensation Reaction

## Experimental Section

### Materials

Dimethylformamide (DMF), acetone, methanol (MeOH), dichloromethane (DCM), and dithiooxamide were purchased from Sigma-Aldrich. Tetrahydrofuran (THF) was purchased commercially from Alfa Aesar. TPE-4Ph-4CHO and TBN-4Ph-4CHO [Schemes S1 and S2] were synthesized by the method mentioned in the previous paper.^52–54^

### Synthesis of TzTz CMPs [TPE-TzTz CMP and TBN-TzTz CMP]

The fabrication of the porous frameworks was executed by a solvothermal condensation strategy. Specifically, a 50 mL flame-dried Schlenk tube was charged with either TPE-4Ph-4CHO (150 mg, 0.2 mmol) or TBN-4Ph-4CHO (150 mg, 0.2 mmol), alongside dithiooxamide (48 mg, 0.4 mmol) in 30 mL of anhydrous DMF. To ensure an oxygen-free environment, the mixture underwent a rigorous degassing sequence before being sealed under a pristine nitrogen atmosphere. The reaction was maintained at a constant temperature of 140 °C with continuous stirring for a duration of 96 h. Upon completion, the system was allowed to equilibrate to room temperature. The resultant crude solid was isolated by filtration and subjected to a stringent washing protocol using a gradient of solvents—DMF, THF, acetone, and methanol—to remove unreacted monomers and entrapped oligomers exhaustively. The final products, TPE–TzTz CMP and TBN–TzTz CMP, were obtained as dark-brown powders with yields exceeding 84% after vacuum drying at 100 °C for 24 h.

### General Photocatalytic Synthesis of 2-Substituted Benzimidazoles

The synthetic route for the preparation of 2-substituted benzimidazoles is illustrated in Scheme 2. In a typical procedure, 1,2-diaminobenzene derivatives (0.2 mmol) and benzaldehyde (0.2 mmol) were dissolved in 5 mL of methanol. To this solution, the CMP catalyst either TPE-TzTz CMP or TBN-TzTz CMP (4 mol %) was added. The resulting suspension was sonicated to ensure homogeneity and subsequently irradiated under 11 W LEDs (positioned at a fixed distance of 7.5 cm) at room temperature for the designated reaction period. The progress of the reaction was monitored via thin-layer chromatography (TLC) using a DCM/acetone (5:1 v/v) mobile phase. Upon quantitative conversion, the heterogeneous catalyst was isolated from the supernatant through centrifugation and subjected to a rigorous washing cycle (3 × 4 mL of methanol) to recover any adsorbed species. The combined organic phases were concentrated under reduced pressure by using a rotary evaporator. The crude residue was purified via silica gel column chromatography to afford the final benzimidazole derivatives. The efficiency of the catalytic system was evaluated based on the isolated mass yields of purified products. Structural integrity and chemical identity were rigorously confirmed using ^1^H NMR and FTIR spectroscopy. The spectral data for the synthesized benzimidazoles align with established references, indicating consistency and reliability in our work. A summary of the structures for all of the obtained benzimidazoles (entries 1–5) is also shown in Scheme 2.

**Scheme 2:**
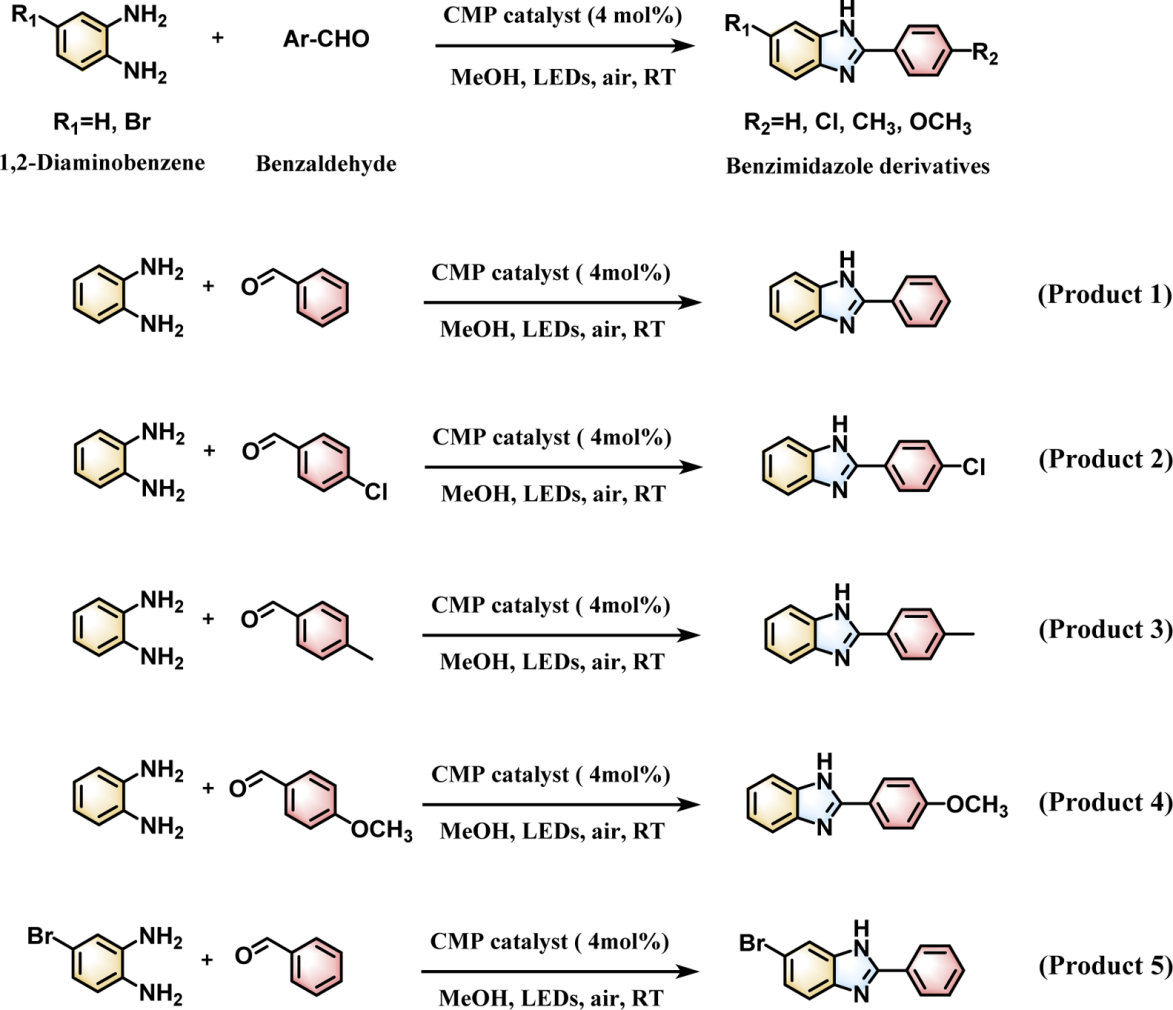
Scope of the Photocatalytic Synthesis of Substituted Benzimidazoles Using TzTz CMPs Catalysts

## Results and Discussion

### Synthesis and Structural Characterization of TPE-TzTz and TBN-TzTz CMPs

The synthesis of TPE-TzTz and TBN-TzTz CMPs [Scheme 1(a) and 1­(b)] was carried out via a high-temperature polycondensation strategy. The selection of monomers was critical: TPE-4Ph-4CHO, featuring a propeller-shaped geometry, or TBN-4Ph-4CHO, a bulky naphthalene-based derivative, served as the primary structural scaffolds. The introduction of TPE and TBN units provides extended π-conjugation and rigid aromatic frameworks, which can enhance the light-harvesting ability and facilitate charge transport within the polymer network. At the same time, the incorporation of the electron-deficient TzTz unit establishes a donor–acceptor (D–A) architecture that promotes efficient charge separation and suppresses recombination of photogenerated charge carriers. These aldehyde monomers [TPE-4Ph-4CHO and TBN-4Ph-4CHO] were coupled with dithiooxamide (DITH) in an anhydrous DMF medium. To ensure a high degree of polymerization and maintain structural integrity, the reaction was conducted in a hermetically sealed Schlenk system under a nitrogen atmosphere. A constant temperature of 140 °C was maintained for 96 h, promoting the irreversible formation of thiazolo­[5,4-*d*]­thiazole (TzTz) linkages, which function as robust connectors within the covalent framework. The characteristic functional groups of TPE-TzTz CMP and TBN-TzTz CMP were analyzed by FTIR spectroscopy, as shown in Figure 1(a) and 1­(b). For the monomer TPE-4Ph-4CHO, characteristic aldehydic absorption bands were observed at 2838 and 2734 cm^–1^ (H–C=O stretching), along with a strong C=O stretching band at 1698 cm^–1^. Similarly, TBN-4Ph-4CHO exhibited aldehydic absorption bands at 2820 and 2724 cm^–1^, and a C=O stretching band was observed at 1704 cm^–1^. The FTIR spectrum of dithiooxamide (DITH) displayed broad absorption bands in the range 3292–3148 cm^–1^, corresponding to the NH_2_ stretching vibrations. After the polycondensation reaction between TPE-4Ph-4CHO and DITH to form TPE-TzTz CMP, the characteristic O=CH peaks of the aldehyde and the NH_2_ bands of DITH were significantly weakened or disappeared, indicating successful condensation, as presented in the FTIR spectrum of TPE-TzTz CMP [Figure 1(a)] and showed new absorption bands at 3023, 1666, 1601, and 820 cm^–1^, which were assigned to aromatic C–H, C=N, C=C, and C–S stretching vibrations, respectively.^55,56^ Likewise, in the synthesis of TBN-TzTz CMP, the characteristic O=CH peaks of TBN-4Ph-4CHO and the NH_2_ bands of DITH were markedly reduced after the reaction, further supporting successful polymer formation. The FTIR spectrum of TBN-TzTz CMP [Figure 1(b)] exhibited absorption peaks at 3018, 1655, 1604, and 816 cm^–1^, corresponding to aromatic C–H, C=N, C=C, and C–S stretching vibrations, respectively. The solid-state ^13^C NMR spectra of TPE-TzTz and TBN-TzTz CMPs [Figure 1(c) and 1­(d)] display characteristic resonances of aromatic carbons at 128.68, 131.18, and 141.83 ppm for TPE-TzTz CMP, and at 129.09, 132.09, and 141.16 ppm for TBN-TzTz CMP, along with signals at 152.91 and 170.31 ppm for TPE-TzTz CMP and 152.65 and 170.63 ppm for TBN-TzTz CMP, which are assigned to C=C and C=N carbon atoms in the TzTz unit, respectively.^57^ To further validate and analyze the chemical environments and bonding topologies of the two TzTz CMPs, the survey spectra obtained from X-ray photoelectron spectroscopy (XPS) reveal that TPE-TzTz and TBN-TzTz CMPs consist mainly of carbon, nitrogen, and sulfur, confirming the successful incorporation of these elements into the TPE-TzTz and TBN-TzTz CMPs frameworks [Figures S1 and S2].

**Figure 1:**
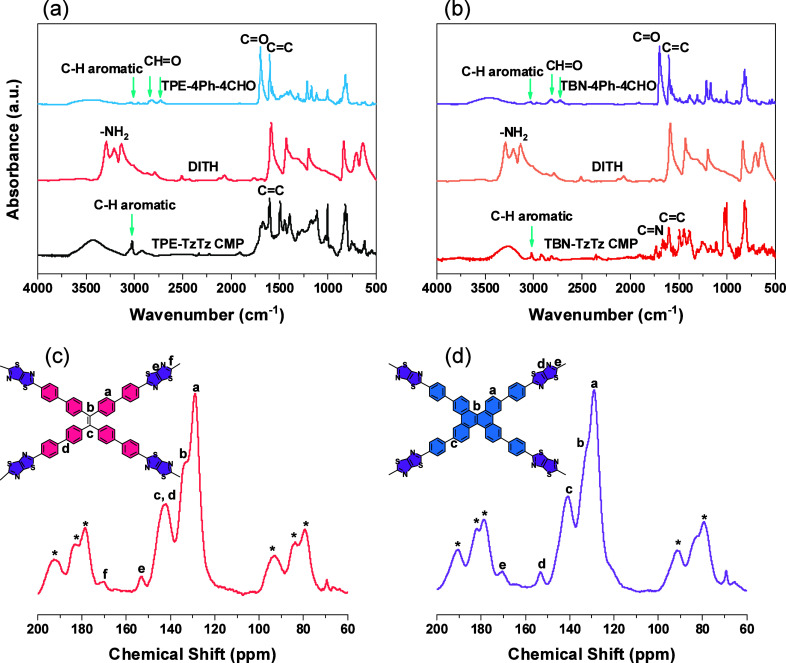
FTIR profiles of (a) TPE-4Ph-4CHO, DITH, and TPE-TzTz CMP and (b) TBN-4Ph-4CHO, DITH, and TBN-TzTz CMP. (c,d) Solid-state ^13^C NMR profiles of (c) TPE-TzTz CMP and (d) TBN-TzTz CMP.

The high-resolution C 1s spectrum of the TPE-TzTz CMP [Figure S3­(a)] could be deconvoluted into three peaks assigned to C–C/C=C (282.8 eV), C–N/C–S (284.1 eV), and C=N/C=S (288.7 eV), respectively. Similarly, the C 1s spectrum of TBN-TzTz CMP [Figure S3­(d)] exhibited three fitted peaks at 282.8, 284.1, and 288.5 eV, corresponding to C–C/C=C, C–N/C–S, and C=N/C=S, respectively.^55,56^ The high-resolution N 1s XPS spectra of both TPE-TzTz and TBN-TzTz CMPs [Figure S3­(b,e)] showed a fitted peak at 397.2 eV, which was attributed to C=N, while the peaks at 399.7 eV (TPE-TzTz CMP) and 399.5 eV (TBN-TzTz CMP) were assigned to C–N.^55,56^ In addition, the fitted S 2p spectra of both TPE-TzTz and TBN-TzTz CMPs [Figure S3­(c,f)] displayed two characteristic peaks at 163.1 and 167.5 eV, corresponding to S 2p_3/2_ and S 2p_1/2_, respectively.^55,56^

The porosities of the two TzTz CMPs were investigated through nitrogen adsorption–desorption measurements at 77 K. As shown in Figure 2(a), TPE-TzTz CMP exhibited a type II isotherm,^58^ characterized by a steep nitrogen uptake at a relative pressure (*P*/*P*_0_) below 0.02, followed by a pronounced increase in adsorption in the high-pressure region (*P*/*P*_0_ = 0.9–1.0), indicating the presence of both micro- and mesoporosity. In contrast, TBN-TzTz CMP displayed a type I isotherm [Figure 2(b)], featuring a sharp nitrogen uptake at *P*/*P*_0_ <0.02, a gradual increase in the range of 0.02–0.85, and a further rise at *P*/*P*_0_ = 0.85–1.0, suggesting a predominantly microporous structure. The specific surface area and total pore volume of the TPE-TzTz CMP were determined to be 484 m^2^ g^–1^ and 1.2 cm^3^ g^–1^, respectively. To gain further insight into the pore architecture, the pore size distributions were analyzed using nonlocal density functional theory (NLDFT). The results revealed that the TPE-TzTz CMP possesses a hierarchical pore structure consisting of both micropores and mesopores, with pore diameters ranging from 1.11 to 1.80 nm [Figure 2(c)]. In comparison, the TBN-TzTz CMP exhibited a specific surface area of 419 m^2^ g^–1^ and a total pore volume of 0.2 cm^3^ g^–1^. NLDFT analysis indicated that TBN-TzTz CMP is mainly composed of micropores, with an average pore diameter in the range of 1.46 to 2.69 nm [Figure 2(d)]. Thermogravimetric analysis (TGA) of the two TzTz-linked CMPs was carried out under a nitrogen atmosphere at a heating rate of 20 °C min^–1^ [Figure 2(e)]. Both materials demonstrated excellent thermal stability, exhibiting 10% weight-loss decomposition temperatures of 508 °C for TPE-TzTz CMP and 483 °C for TBN-TzTz CMP. In addition, high char yields of 70 and 69 wt % were retained for TPE-TzTz and TBN-TzTz CMPs, respectively, further confirming their strong thermal resistance and structurally stable carbon frameworks. The porosity and thermal stability properties of the synthesized TPE-TzTz and TBN-TzTz CMPs are summarized in Tables S1 and S2. The powder X-ray diffraction (PXRD) patterns presented in Figure 2(f) reveal the absence of sharp diffraction peaks, with only a broad halo centered at around 18° observed for both materials. This indicates a lack of long-range crystalline order, confirming that both TPE-TzTz and TBN-TzTz CMPs are predominantly amorphous in nature.

**Figure 2:**
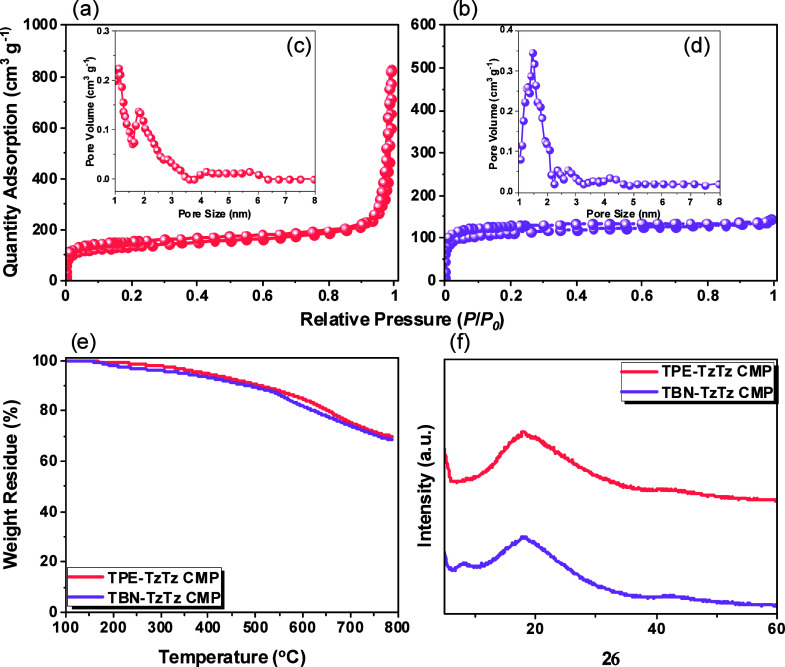
N_2_ adsorption–desorption isotherms (a,b) and pore size distributions (c,d) of (a,c) TPE-TzTz and (b,d) TBN-TzTz CMPs. TGA curves (e) and PXRD patterns (f) of TPE-TzTz and TBN-TzTz CMPs.

Figure 3(a) shows the SEM image of the TPE-TzTz CMP, which exhibits irregularly clustered spherical aggregates. In contrast, the particles observed in TBN-TzTz CMP [Figure 3(b)] are noticeably smaller than those in TPE-TzTz CMP. The prediction of particle size and morphology in microporous materials synthesized via solvothermal methods remains challenging due to the complexity of the nucleation and growth processes. We propose that the relatively stronger π–π stacking interactions in TBN-TzTz CMP, compared to TPE-TzTz CMP, contribute to the formation of smaller particles.^59^ The amorphous nature and lack of long-range order in both TPE-TzTz and TBN-TzTz CMPs were further confirmed by transmission electron microscopy (TEM), as shown in Figure 3(c) and 3­(d), which are consistent with the PXRD results. Furthermore, energy-dispersive X-ray spectroscopy (EDS) analysis [Figure 3(e) and 3­(f)] verifies the presence of aromatic carbon (violet), nitrogen (green), and sulfur (red) atoms in both TzTz-linked CMP frameworks, supporting their proposed chemical compositions.

**Figure 3:**
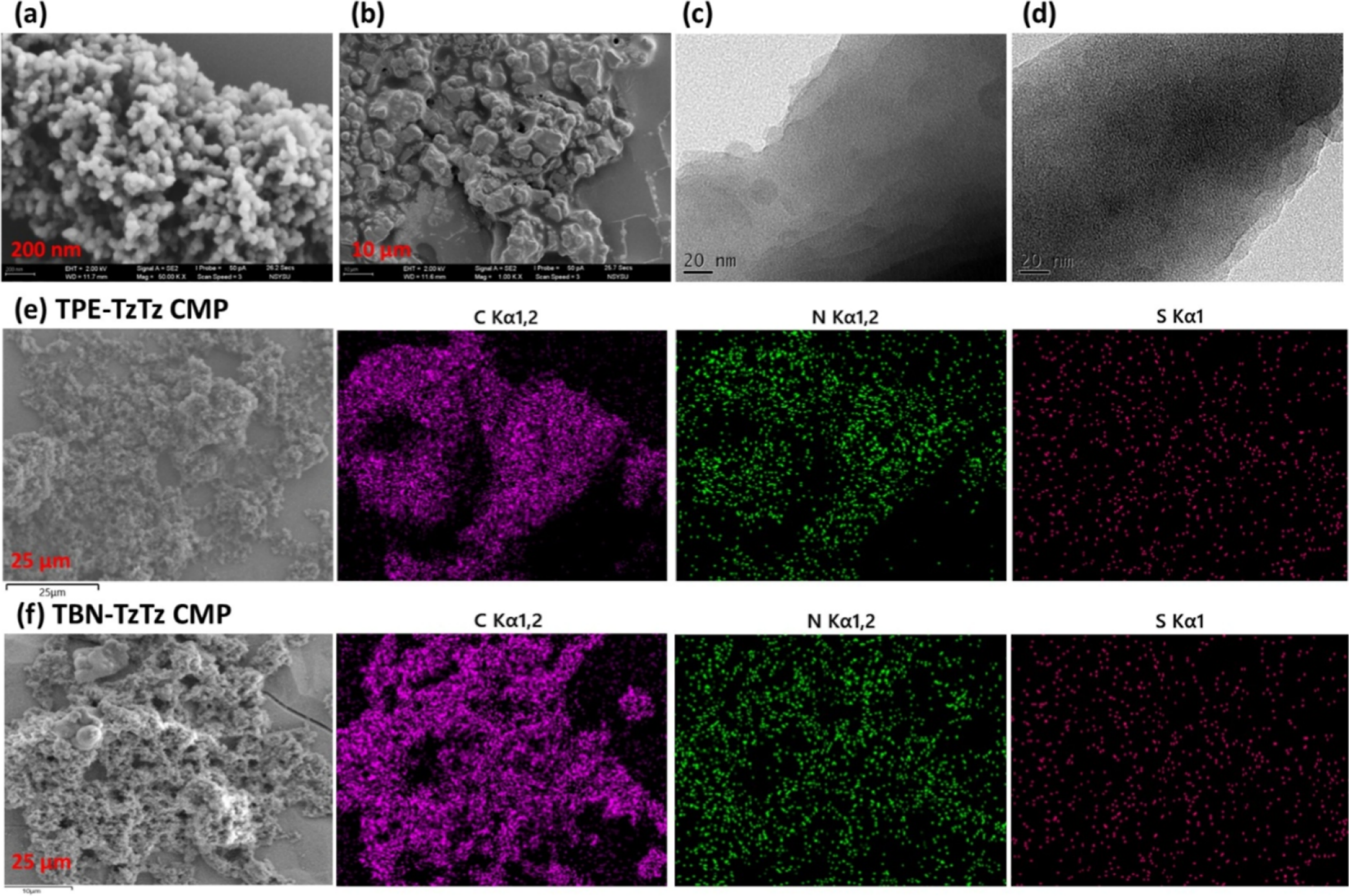
(a,b) SEM images and (c,d) TEM images of (a,c) TPE-TzTz and (b,d) TBN-TzTz CMPs. (e,f) SEM-EDS mapping of (e) TPE-TzTz and (f) TBN-TzTz CMPs.

UV–Vis diffuse reflectance spectroscopy (DRS) was performed on all TzTz-linked CMPs. As shown in Figure 4(a), both TPE-TzTz and TBN-TzTz CMPs exhibited strong absorption in the visible-light region with a maximum absorption peak at 426.5 nm. Notably, the two samples displayed very similar absorption profiles. The optical band gap energies of TPE-TzTz and TBN-TzTz CMPs were determined from their respective Tauc plots [Figure 4(b)] and were calculated to be 2.48 eV for TPE-TzTz CMP and 2.42 eV for TBN-TzTz CMP. To further evaluate the energy levels of the TPE-TzTz and TBN-TzTz CMPs, valence band X-ray photoelectron spectroscopy (VB-XPS) measurements were conducted. As illustrated in Figure 4(c), the valence band (VB) edge positions were determined to be 1.32 eV for TPE-TzTz CMP and 0.91 eV for TBN-TzTz CMP. Based on these VB values and the corresponding optical band gap energies, the conduction band (CB) potentials were calculated to be −1.16 eV for TPE-TzTz CMP and −1.51 eV for TBN-TzTz CMP, as shown in Figure 4(d).

**Figure 4:**
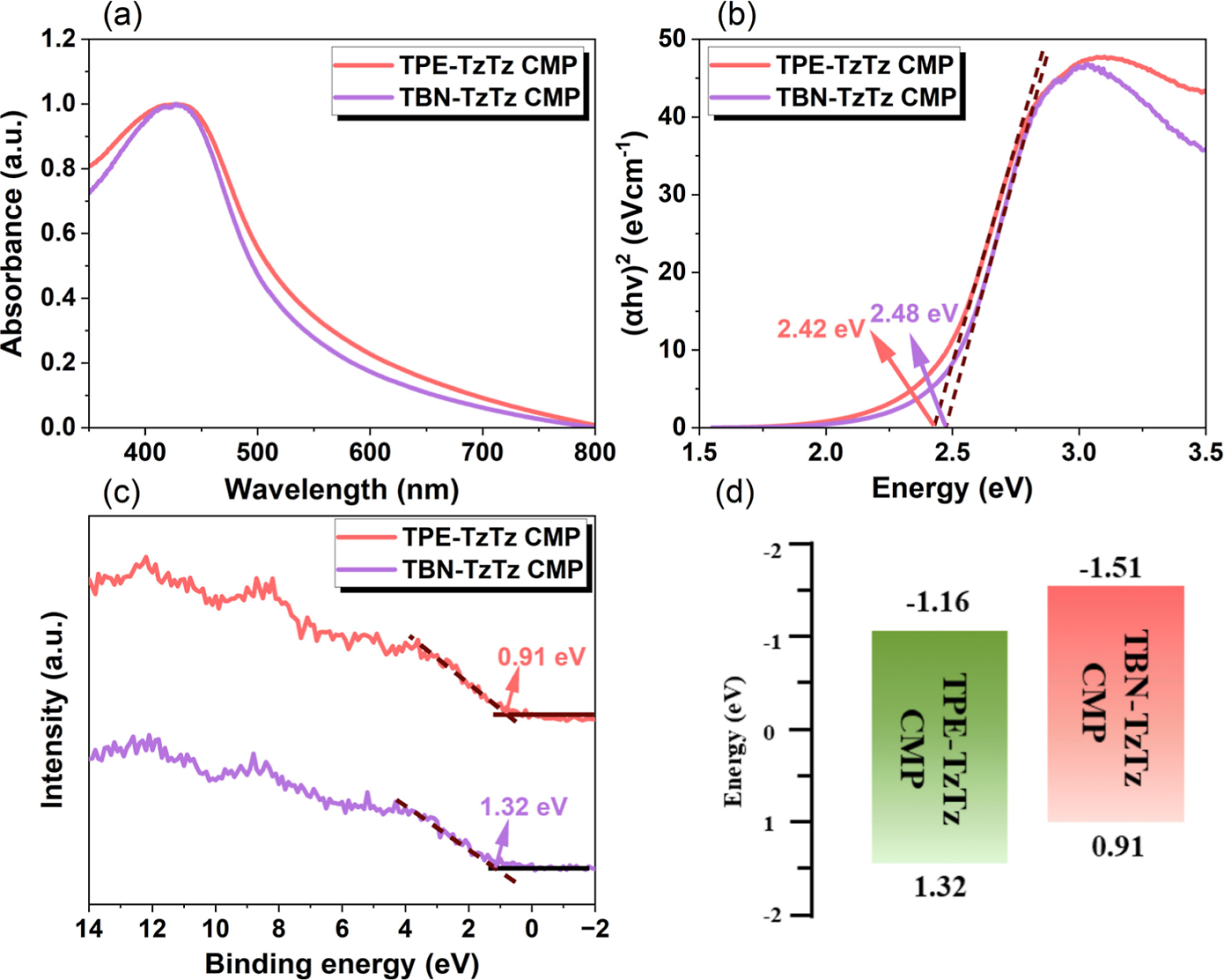
(a) UV–Vis diffuse reflectance spectroscopy, (b) Tauc plots, (c) VB-XPS profiles, and (d) energy diagram-level potentials of TPE-TzTz CMP and TBN-TzTz CMP.

Furthermore, the calculated CB potentials suggest that the photoinduced reduction of O_2_ to superoxide radicals (O_2_^•–^, −0.33 eV vs NHE) is thermodynamically feasible. This indicates that upon light irradiation, electrons from the excited TzTz-linked CMPs can be effectively transferred to the O_2_ molecules, facilitating the formation of reactive oxygen species. The reactive materials TPE-TzTz CMP and TBN-TzTz CMP effectively promote the formation of a blue 1,4-bis­(diphenylamino)­benzene cationic radical and superoxide under light irradiation, as shown in Figure 5(a). This process is mediated by electron transfer from *N*,*N*,*N*′,*N*′-tetramethyl-*p*-phenylenediamine (TMPD) to molecular oxygen. The strong π–π stacking interactions in the TzTz-linked CMPs facilitate efficient electronic transmission along the conjugated π-skeletons. Notably, the deeper blue color and higher absorbance of the cationic radical indicate that TBN-TzTz CMP exhibits superior photocatalytic activity compared to TPE-TzTz CMP. Under light illumination, both TPE-TzTz and TBN-TzTz CMPs displayed rapid and reproducible photocurrent responses during multiple on–off irradiation cycles, providing clear evidence of photoinduced charge carrier transfer within these materials [Figure 5(b)]. In particular, TBN-TzTz CMP showed a significantly stronger transient photocurrent than TPE-TzTz CMP, suggesting more efficient separation of photogenerated electron–hole pairs. Furthermore, the stable cycle-to-cycle response demonstrates excellent photostability, while the gradual increase in photocurrent suggests that a short initial conditioning period under light may be required to achieve optimal performance. 5,5-Dimethyl-1-pyrroline-*N*-oxide (DMPO) was employed as a spin-trapping agent in electron spin resonance (ESR) measurements to detect superoxide formation in the TzTz-linked CMPs. Characteristic ESR signals corresponding to O_2_^•–^ were observed under visible-light irradiation [Figure 5(c) and 5­(d)], confirming that superoxide radicals were successfully generated in both TPE-TzTz and TBN-TzTz CMPs. Electrochemical impedance spectroscopy (EIS) was employed to evaluate the charge transfer characteristics and interfacial properties of TPE-TzTz and TBN-TzTz CMPs [Figure S4], and the Nyquist plots and equivalent Randles circuit model were used to calculate the charge transfer resistance (*R*_ct_) and solution resistance (*R*_s_) of TPE-TzTz CMP and TBN-TzTz CMP. The calculated *R*_ct_ values of TBN-TzTz CMP and TPE-TzTz CMP are 7 and 45 Ω, respectively, which indicates that the TBN-TzTz CMP facilitates charge transfer in a more efficient way than the TPE-TzTz CMP. The *R*_s_ values of TBN-TzTz CMP and TPE-TzTz CMP were 10.8 and 11.3 Ω; respectively.

**Figure 5:**
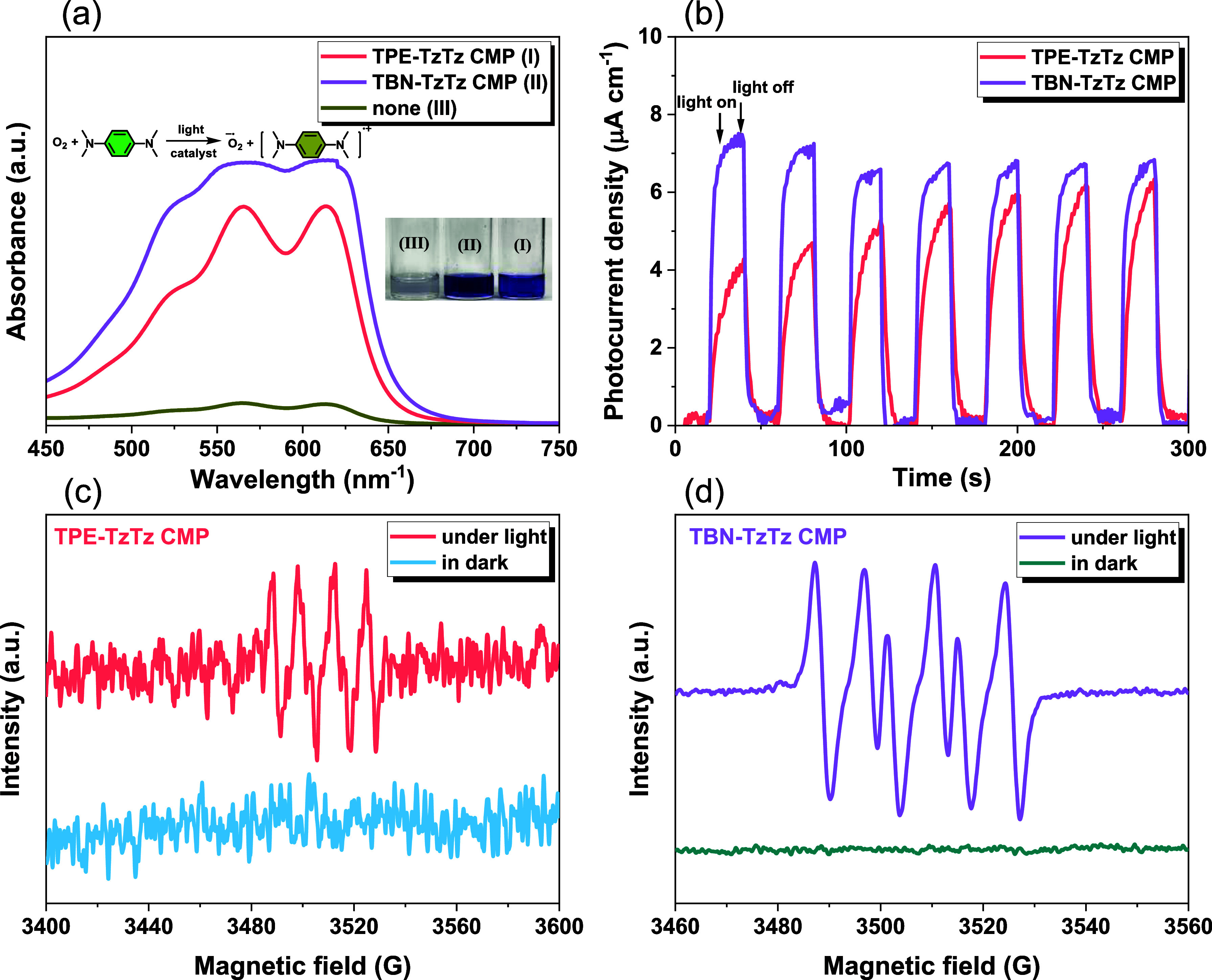
(a) UV–Vis absorbance spectroscopy and (b) photocurrent plots of TPE-TzTz CMP and TBN-TzTz CMP. (c,d) ESR measurements profiles of (c) TPE-TzTz and (d) TBN-TzTz CMPs.

These findings indicate that *R*_s_ has a negligible influence on the observed differences. Therefore, the markedly lower *R*_ct_ is the dominant contributor to the superior charge transfer kinetics of the TBN-TzTz CMP. The TPE-TzTz and TBN-TzTz CMPs exhibit intrinsic porosity, excellent stability, and favorable photoredox properties, enabling efficient light harvesting in the visible region. These features render them highly suitable as heterogeneous photocatalysts. After optimizing the reaction conditions, we investigated the reaction scope to evaluate the photocatalytic performance of the TzTz-linked CMPs for all the obtained benzimidazoles (entries 1–5) as shown in Scheme 2, and the results are summarized in Table 1. We carried out a photocatalytic synthesis of benzimidazole using 1,2-diaminobenzene, benzaldehyde, and molecular oxygen from air as substrates with an environmentally benign oxidant.

**Table 1:** Substrate Scope of TzTz-Linked-CMP-Catalyzed Benzimidazole Synthesis

entry^a^	catalyst^b^	product	solvent^c^	lamp^d^	time (h)	yield (%)
1	TPE-TzTz CMP	product 1	MeOH	blue	2.5	88
2	TBN-TzTz CMP	product 1	MeOH	blue	2	90
3	TPE-TzTz CMP	product 2	MeOH	blue	2.5	83
4	TBN-TzTz CMP	product 2	MeOH	blue	2	85
5	TPE-TzTz CMP	product 3	MeOH	blue	2.5	87
6	TBN-TzTz CMP	product 3	MeOH	blue	2	88
7	TPE-TzTz CMP	product 4	MeOH	blue	4	80
8	TBN-TzTz CMP	product 4	MeOH	blue	3.5	89
9	TPE-TzTz CMP	product 5	MeOH	blue	3.5	85
10	TBN-TzTz CMP	product 5	MeOH	blue	3	82
11	TPE-TzTz CMP	product 1	MeOH	white	5	86
12	TBN-TzTz CMP	product 1	MeOH	white	5.5	84
13	none	product 1	MeOH	blue	8	trace
14	TPE-TzTz CMP	product 1	MeOH	none	8	trace
15	TPE-TzTz CMP	product 1	THF	blue	8	trace
16	TPE-TzTz CMP	product 1	DMF	blue	8	trace
17	TPE-TzTz CMP	product 1	EtOH	blue	3	83

a 1,2-Diaminobenzene (0.2 mmol), benzaldehyde (0.2 mmol).

b TzTz-linked-CMP catalyst (4 mol %).

c Solvent (4.0 mL).

d 11 W LEDs, air, room temperature. Isolated yields after column chromatography.

Under the optimized reaction conditions, the photocatalytic activity of the newly developed TPE-TzTz and TBN-TzTz CMPs was assessed. Upon irradiation with 11 W blue LEDs in methanol for 2 to 4 h, high product yields of 90% and 80% were obtained for the TzTz-linked CMPs (Table 1, entries 1–10). The characterization data of these catalytic products are provided in Figures S5–S14.^60–66^

The superior catalytic performance of TBN-TzTz CMP compared with TPE-TzTz CMP can be attributed to stronger π–π stacking interactions and more favorable energy band structures within the TzTz-linked frameworks. When a white lamp was used as the light source, slightly lower yields of 86% and 84% were obtained (Table 1, entries 11 and 12), highlighting the importance of light wavelength. We further examined the influence of different solvents on the reaction efficiency. Control experiments confirmed that both the presence of the photocatalyst and light irradiation were essential for the reaction to proceed (Table 1, entries 13 and 14). In aprotic solvents, such as DMF and THF, significantly reduced yields were observed (Table 1, entries 15 and 16). In contrast, EtOH resulted in a high yield of 83% under identical conditions (Table 1, entry 17), demonstrating its suitability as a reaction medium for this photocatalytic system. The photocatalytic transformation is initiated by a condensation reaction between 1,2-diaminobenzene and benzaldehyde (I), as illustrated in Figure 6, resulting in the formation of a Schiff base intermediate (II). This imine subsequently undergoes intramolecular cyclization to generate intermediate (III), which features an electron-rich benzimidazole precursor structure. Upon visible-light irradiation, the TzTz-linked CMPs are photoexcited to a higher energy state (TzTz-linked CMPs*). The excited photocatalyst then participates in a reductive quenching process by accepting an electron from intermediate (III) via a single-electron transfer, producing a radical intermediate (IV) and the reduced photocatalyst (TzTz-linked CMP^•–^). The reduced CMP^•–^ species is subsequently oxidized by molecular oxygen (O_2_), generating superoxide anion radicals (O_2_^•–^) while regenerating the ground-state CMP photocatalyst, thereby completing the photocatalytic cycle. The resulting reactive oxygen species, including superoxide (O_2_^•–^) and hydroperoxyl radicals (HOO^•–^), play a crucial role in driving the reaction forward. These species facilitate key deprotonation and oxidative dehydrogenation steps that ultimately lead to the formation of the benzimidazole product (V). In addition, further transformations involving radical species such as HOO^•–^ and H_2_O_2_ may also contribute to the reaction pathway or influence product stabilization. After the first reaction cycle, the TPE-TzTz and TBN-TzTz CMP photocatalysts were recovered by centrifugation and thoroughly washed with H_2_O and THF to ensure complete removal of residual reactants. The recovered TzTz-linked CMPs were then dried under reduced pressure at 110 °C for 1 day before being reused in the subsequent photocatalytic reaction. This recovery and purification procedure was repeated after each cycle. The FTIR spectra (Figure S15­(a,b), TEM images (Figure S15­(c,e,g,i)), and SEM images (Figure S15­(d,f,h,j)] of the TzTz-linked CMPs showed no noticeable changes throughout the cycles. In addition, no significant variation in reaction yields was observed between the cycles.

**Figure 6:**
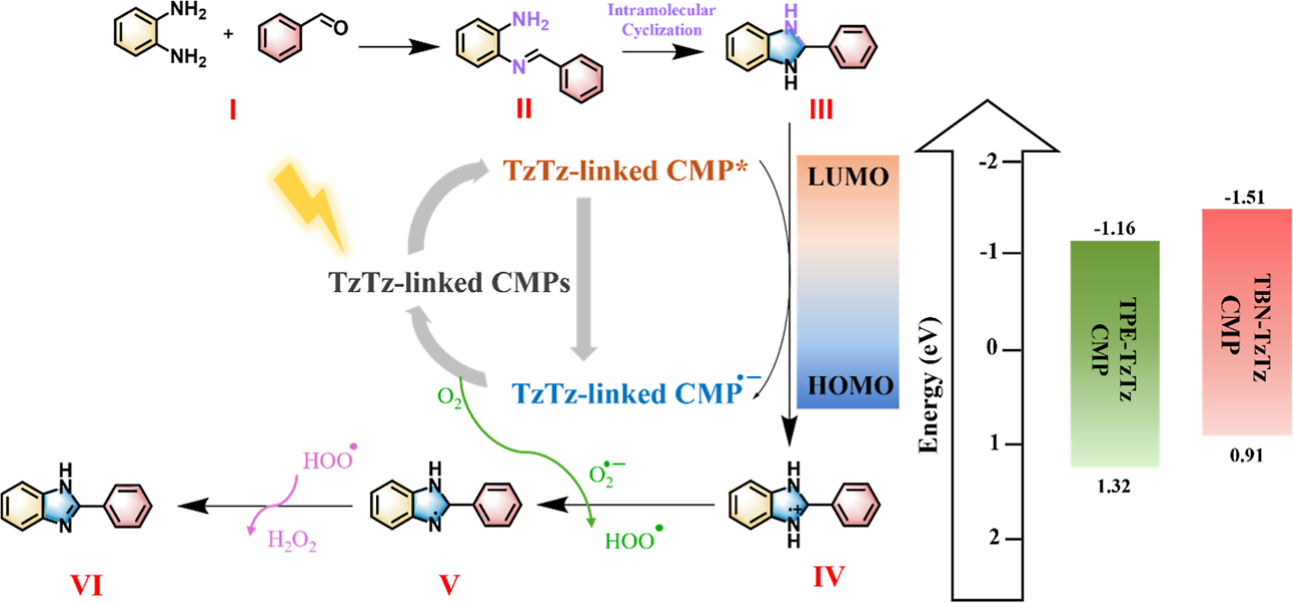
Schematic depiction of the benzimidazole synthesis mechanism under visible-light irradiation with TzTz-linked-CMPs.

To gain deeper insight into the photocatalytic performance of our synthesized TPE-TzTz and TBN-TzTz CMP photocatalyst materials, we compared the benzimidazole yields obtained using various reported heterogeneous photocatalysts, as summarized in Figure 7.^67–76^ Both TPE-TzTz and TBN-TzTz CMPs display excellent catalytic performance, achieving yields of approximately 85%, which clearly surpass those of many conventional inorganic or hybrid photocatalysts. Notably, TPE-TzTz and TBN-TzTz CMPs photocatalysts featuring D–A architectures are predominantly clustered in the high-yield region, indicating their superior efficiency. In contrast, materials incorporating TPE with a donor–acceptor (D–A) structure, such as PAF-364 and PAF-365,^76^ despite their structural robustness, exhibit comparatively lower photocatalytic activity with yields in the range of 60–70%. This comparative analysis underscores the crucial role of D–A architectures in facilitating effective charge separation and enhancing photocatalytic performance under visible-light irradiation.

**Figure 7:**
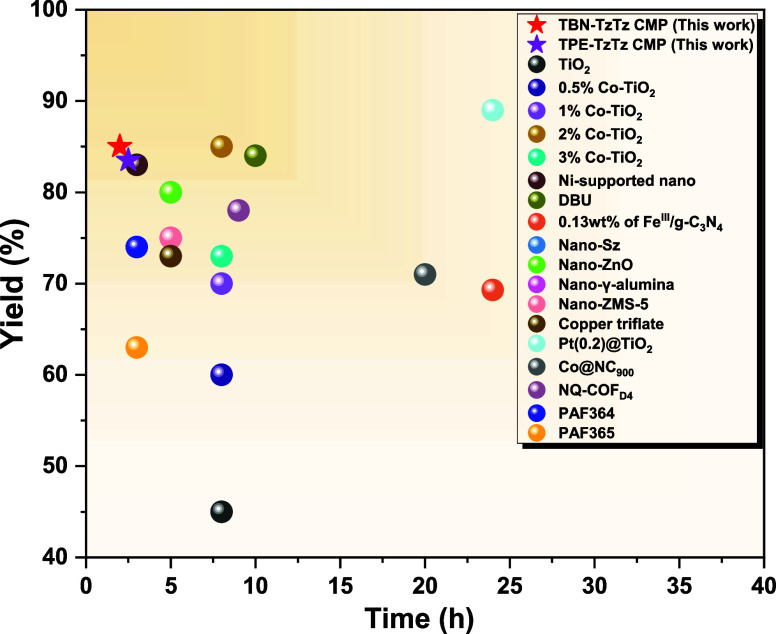
Comparison of photocatalytic performance of TzTz-linked CMPs [TPE-TzTz and TBN-TzTz CMPs] and reported photocatalysts for the synthesis of benzimidazoles.

The MESP mapping is a valuable tool for understanding the distribution of electrostatic potential across entire molecules, which further helps explain how different molecules interact with each other and with light—an important aspect in the rational design of efficient photocatalysts. As shown in Figure 8(a), the MESP maps of TPE-TzTz and TBN-TzTz CMPs display distinct regions of charge separation, with green areas representing regions of intermediate electrostatic potential. This charge separation is significant because it can help reduce charge recombination during photocatalytic processes. Notably, regions of high electron density (indicated by red) are mainly localized around the nitrogen atoms in the TzTz units, suggesting that these sites function as electron-donating centers that are essential for initiating photocatalytic activity. In the TBN-TzTz CMP, a larger region of high electron density is also observed at the central core (shown in yellow), indicating a more electron-rich domain compared to TPE-TzTz CMP. This implies that the central framework in the TBN-TzTz CMP may serve as an additional and stronger electron donor, potentially contributing to improved photocatalytic performance. Overall, the MESP analysis suggests that the TzTz moiety plays a key role as an electron-donating unit, facilitating charge transfer processes that are crucial for effective photocatalysis. The electronic properties of CMPs, particularly the highest occupied molecular orbital (HOMO) and the lowest unoccupied molecular orbital (LUMO), are strongly influenced by their atomic framework and overall molecular architecture. The HOMO is mainly associated with a molecule’s ability to donate electrons, whereas the LUMO reflects its ability to accept electrons. The photocatalytic activity and stability of a material are closely related to the energy gaps between these two orbitals. Figure 8(b) shows the HOMO–LUMO distributions and their corresponding energy levels for TPE-TzTz and TBN-TzTz CMPs. For the TPE-TzTz CMP, the HOMO is delocalized across the entire structure, extending from the central core to the peripheral arms and including the heteroatoms within the TzTz moiety. In contrast, the HOMO in the TBN-TzTz CMP is mainly localized at the central core, consistent with the electron-rich regions observed in the MESP analysis. This central localization suggests a higher electron density at the core, which may enhance its electron-donating ability during photocatalysis. The LUMOs exhibit different spatial distributions compared with the HOMO in both materials, indicating a clear separation between electron-donating and electron-accepting regions. Such spatial separation is beneficial for photocatalysis because it can suppress charge recombination and thus improve the photocatalytic efficiency. Furthermore, the calculated band gap of TBN-TzTz CMP (3.05 eV) is slightly narrower than that of TPE-TzTz CMP (3.17 eV), suggesting that TBN-TzTz CMP requires less energy for electronic excitation. This smaller HOMO–LUMO gap facilitates more efficient charge transfer, which can enhance photocatalytic performance. These results are consistent with experimental observations and indicate that extending π-conjugation in the central framework effectively promotes electron delocalization and reactivity.

**Figure 8:**
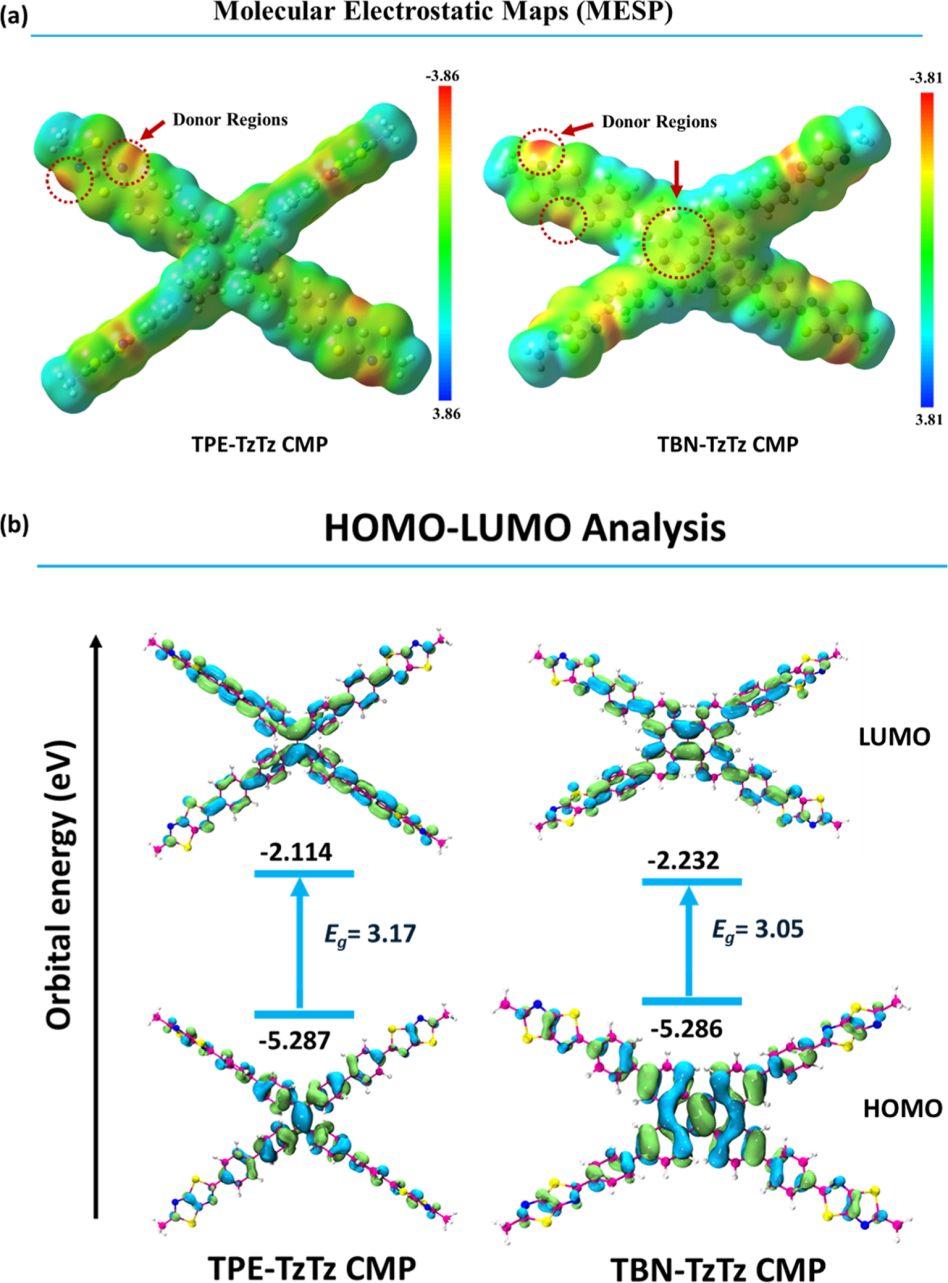
(a) The ESP maps for designed TPE-TzTz and TBN-TzTz CMPs and (b) the visual description of HOMO–LUMO and their associated energies for TPE-TzTz and TBN-TzTz CMPs.

Therefore, rational structural design of TzTz CMPs to fine-tune their electronic properties represents a promising strategy for optimizing the photocatalytic performance. Time-dependent density functional theory (TD-DFT), originally developed by Runge and Gross in 1984,^77^ extends conventional DFT to accurately describe the excited-state electronic properties of molecules and materials.^78^ By modeling the electronic response to time-dependent external perturbations, such as electromagnetic radiation, TD-DFT allows reliable prediction of electronic excitations and photoinduced processes.^79^ In this work, TD-DFT calculations were carried out for both TzTz CMPs, and their simulated absorption spectra are presented in Figure S16. TPE-TzTz exhibits a main absorption peak in the range 400–430 nm, corresponding to a transition energy of approximately 2.78 eV. In comparison, TBN-TzTz CMP exhibits a red-shifted absorption band between 430 and 450 nm, with a slightly lower transition energy of 2.71 eV. The red shift observed for TBN-TzTz CMP indicates absorption at longer wavelengths, reflecting a narrower optical band gap relative to TPE-TzTz CMP. This finding is consistent with the band gap values derived from the HOMO–LUMO analysis. A smaller band gap enables TBN-TzTz CMP to absorb a broader range of visible light, thereby enhancing its potential for photocatalytic applications under visible or solar light irradiation.^80^ Overall, these results demonstrate the superior light-harvesting capability of the TBN-TzTz CMP, as supported by both UV–Vis spectra and TD-DFT calculations. The enhanced absorption and narrower band gap contribute to its improved photocatalytic efficiency and more effective utilization of incident light energy. Notably, the simulated absorption results are in excellent agreement with the experimentally measured UV–Vis spectra. The comparison between experimental observations and computational analyses provides valuable insight into the photocatalytic behavior of the two TzTz-CMPs. DFT calculations indicate that the TBN-TzTz CMP possesses a more favorable electronic structure, characterized by enhanced π-conjugation and a more localized electron density distribution around the central donor unit. Such an electronic configuration facilitates efficient charge delocalization and promotes electron transfer across the conjugated network. Experimentally, this behavior is evidenced by the enhanced photocurrent response and reduced charge transfer resistance observed for the TBN-TzTz CMP, indicating a more efficient charge separation and migration within the framework. These electronic advantages are further supported by the catalytic experiments, where the TBN-TzTz CMP demonstrated faster reaction kinetics and slightly higher yields in the photocatalytic synthesis of benzimidazole derivatives. Although the DFT calculations were performed by using simplified models based on monomeric units of the polymeric framework, which may not fully reproduce the absolute experimental values, the predicted trends are in good agreement with the experimental results. This consistency suggests that the superior photocatalytic performance of the TBN-TzTz CMP can be attributed to its more efficient charge transport and improved electronic communication within the donor–acceptor architecture, as reflected by the HOMO–LUMO distributions [Figure 8(b)].

## Conclusions

In summary, two CMPs incorporating TzTz units [TPE-TzTz and TBN-TzTz CMPs] were synthesized, featuring donor–acceptor (D–A) architectures, one based on TPE and the other on TBN. These structures facilitate effective separation and transport of photoinduced charge carriers, significantly enhancing their catalytic performance under visible-light irradiation. Both CMPs exhibited remarkable photocatalytic activity for the synthesis of benzimidazoles stemming from their strong ability to generate and transfer photogenerated charge carriers and superoxide radicals. The results confirmed that TzTz-linked CMPs efficiently catalyze benzimidazole formation under both blue and white light in the air. Notably, the TBN-TzTz CMP demonstrated superior photocatalytic activity compared to the TPE-TzTz CMP. This enhancement is likely due to the higher planarity and rigidity of the TBN-TzTz CMP, which promotes stronger π–π stacking and facilitates charge carrier transport. Importantly, the heterogeneous nature of these photocatalysts allows for facile recovery and reuse, aligning with sustainable development. After the catalytic cycles, the recycled TzTz-linked CMPs retained their structural integrity, as evidenced by consistent FTIR spectra and preserved morphology, underscoring their excellent stability. These results highlight the promise of TzTz-linked CMPs as efficient, recyclable, and stable heterogeneous photocatalysts for visible-light-induced organic transformations.

## Supplementary Material



## Notes

Instrumentation; computational methods; synthesis details of benzimidazole products; synthesis method of TPE-4Ph-4CHO and TBN-4Ph-4CHO; XPS survey spectrum of TPE-TzTz CMP and TBN-TzTz CMP; EIS data of TzTz-linked CMPs; summarized BET parameters; thermal stability of the as-synthesized TPE-TzTz CMP and TBN-TzTz CMP; FTIR and ^1^H NMR results of benzimidazole derivatives; stability data including FTIR, TEM, and SEM of TPE-TzTz CMP and TBN-TzTz CMP after photocatalytic reaction; and UV–vis Spectra of TPE-TzTz and TBN-TzTz CMPs (PDF)
