# Supplementary material for: Metal-Free Tetraphenylethylene and Dibenzo[g,p]chrysene with Thiazolo[5,4‑d] Thiazole-Based Donor–Acceptor Conjugated Microporous Polymers for the Efficient Photocatalytic Synthesis of 2‑Substituted Benzimidazole
Source: ACS Polym Au. 2026 Mar 16;6(3):927–40. doi: 10.1021/acspolymersau.6c00023 (PMC13261737; doi:10.1021/acspolymersau.6c00023)
Supplement: Supplementary file 1 [file lg6c00023_si_001.pdf]

Supporting Information

Metal-Free Tetraphenylethylene and Dibenzo[g,p]chrysene with Thiazolo[5,4-d] Thiazole-Based Donor-Acceptor Conjugated Microporous Polymers for the Efficient Photocatalytic Synthesis of 2-Substituted Benzimidazole

Mohamed Gamal Mohamed,[a,b]* Pei-Tzu Wang,[a] Hira Karim,[c] and Shiao-Wei Kuo[a,]*

[a]Department of Materials and Optoelectronic Science, Center for Functional Polymers and Supramolecular Materials, National Sun Yat-Sen University, Kaohsiung 804, Taiwan.

[b]Chemistry Department, Faculty of Science, Assiut University, Assiut 71515, Egypt.

[c]Department of Chemistry, School of Natural Sciences (SNS), National University of Sciences and Technology (NUST), H-12, Islamabad, 44000, Pakistan.

***Corresponding authors**: mgamal.eldin34@gmail.com (M. G. Mohamed) and kuosw@faculty.nsysu.edu.tw (S.-W. Kuo).

Characterization

FTIR measurements were performed on a Bruker Tensor 27 spectrometer at a resolution of 4 cm^{-1} with the KBr pellet technique. Raman spectra were recorded using a HORIBA iHR550 spectrometer equipped with a 532 nm laser excitation source and a CCD detector. The measurements were conducted at room temperature with a spectral resolution of 1 cm^{-1}. For solution-state nuclear magnetic resonance (NMR), spectra were recorded on an INOVA 500 MHz system using DMSO-d_6 and $CDCl_3$ as solvents, and TMS as the external standard; chemical shifts are reported in ppm. Thermal analysis was conducted on a TG Q-50 under nitrogen flow (60 mL min^{-1}) with samples (ca. 6 mg) heated in a platinum pan from 100 to 800 °C at 20 °C min^{-1}. Solid-state ^{13}C NMR spectra were obtained using a JEOL JNM-LA300 spectrometer with a CP/MAS probe operating at 75.577 MHz. Polymer network morphology was characterized by field-emission scanning electron microscopy (FE-SEM; JEOL JSM7610F). Surface area and porosity measurements were carried out using a BEL MasterTM/BEL simTM (version 3.0.0) system, with sample masses in the range of 40–60 mg. Nitrogen (N_2) adsorption–desorption isotherms were recorded at 77 K by incrementally exposing the samples to ultrahigh-purity N_2 gas, with pressures gradually increasing up to approximately 1 atm. The measurements were conducted in a liquid nitrogen bath to maintain a constant temperature. Before testing, the samples were degassed under vacuum at 150 °C for 8 hours to remove adsorbed species. BET theory was applied for surface area evaluation, while pore size distribution was derived using the nonlocal density functional theory (NLDFT). X-ray diffraction (XRD) patterns were obtained using a Bruker D2-Phaser, and surface elemental composition was analyzed via X-ray photoelectron spectroscopy (XPS, ULVAC-PHI Quantes). Electrochemical analyses were conducted using a ZAHNER ZENNIUM impedance measurement analyzer. The UV–Vis absorption spectra of the synthesized TztTz CMPs

were recorded using a Hitachi U-3300 spectrophotometer with a concentration of 0.4 mg mL^{-1} in N-methyl pyrrolidone (NMP). Electron paramagnetic resonance (EPR) measurements were carried out on an EMXnano bench-top system after dispersing the samples in methanol at room temperature for 10 minutes.

The synthesis of benzimidazole products

Synthesis of 2-Phenylbenzimidazole (Product 1): This process resulted in the isolation of the product as a white solid. FTIR [**Figure S5**]: 3047, 1622, 1591, 1444, 1277, 1118, and 687 cm^{-1}. ^1H NMR (600 MHz, DMSO-d_6) [**Figure S6**]: 12.91 (br, 1H), 8.17 (d, J = 7.2 Hz, 2H), 7.66-7.54(m, 5H), 7.23-7.20 (m, 2H) ppm.

Synthesis of 2-(4-Chlorophenyl)benzimidazole (Product 2): FTIR [**Figure S7**]: 3047, 1622, 1591, 1444, 1277, 1118, and 687 cm^{-1}. ^1H NMR (600 MHz, DMSO-d_6) [**Figure S8**]: 12.91 (br, 1H), 8.17 (d, J = 7.2 Hz, 2H), 7.66-7.54(m, 5H), 7.23-7.20 (m, 2H) ppm.

Synthesis of 2-(4-Tolyl)benzimidazole (Product 3): FTIR [**Figure S9**]: 2961, 2918, 2850, 1586, 1500, 1430, 1274, 1226, 964, 820, 748, and 544 cm^{-1}. ^1H NMR (600 MHz, DMSO-d_6) [**Figure S10**]: 12.82 (bs, 1H), 8.08-8.05 (m, 2H), 7.63-7.05 (m, 2H), 7.36-7.34 (m, 2H), 7.36-7.20 (m, 2H), 2.38 (s, 3H) ppm.

Synthesis of 2-(4-Methoxyphenyl)benzimidazole (Product 4): FTIR [**Figure S11**]: 3051, 2926, 2804, 2670, 1610, 1501, 1437, 1250, 1180, 1030, 745, and 549 cm^{-1}. ^1H NMR (600 MHz, DMSO-d_6) [**Figure S12**]: 12.75 (br, 1H), 8.15-8.11 (m, 2H), 7.63-7.62 (m, 1H), 7.51-7.49 (m, 1H), 7.19-7.16 (m, 2H), 7.14-7.10 (m, 2H), 3.85 (s, 3H) ppm.

Synthesis of 2-(4-Methoxyphenyl)benzimidazole (Product 5): The FTIR [**Figure S13**]: 3051, 2926, 2804, 2670, 1610, 1501, 1437, 1250, 1180, 1030, 745, and 549 cm^{-1}. ^1H NMR (600 MHz, DMSO-d_6) [**Figure S14**]: 13.11 (br s, 1H), 8.17 (d, J = 8.0 Hz, 2H), 7.69-7.79 (m, 1H), 7.56-7.63 (m, 4H), 7.35 ppm (d, J = 8.4 Hz, 1H) ppm.

First-Principles approach towards Photocatalysts

To investigate the nature of the donor-acceptor pattern in the framework of synthesized TzTz CMPs and their respective molecular orbital energies, DFT calculations were carried out [**1**]. All the structures investigated were optimized at B3LYP/6-31G (d, p) [**2,3**] using the Gaussian 09 software package [**4**] with the imaginary frequency confirmed to be zero. To get better insight into locating interaction regions on the studied structures, Molecular Electrostatic Potential (MESP) maps were generated. Also, to understand the location of frontier molecular orbitals, HOMO-LUMO analysis was conducted using Multiwfn [**5**] and the Visual Molecular Dynamics (VMD) program [**6**]. Also, Time-dependent DFT (TD-DFT) calculations were performed to assess the charge transition at excited states.

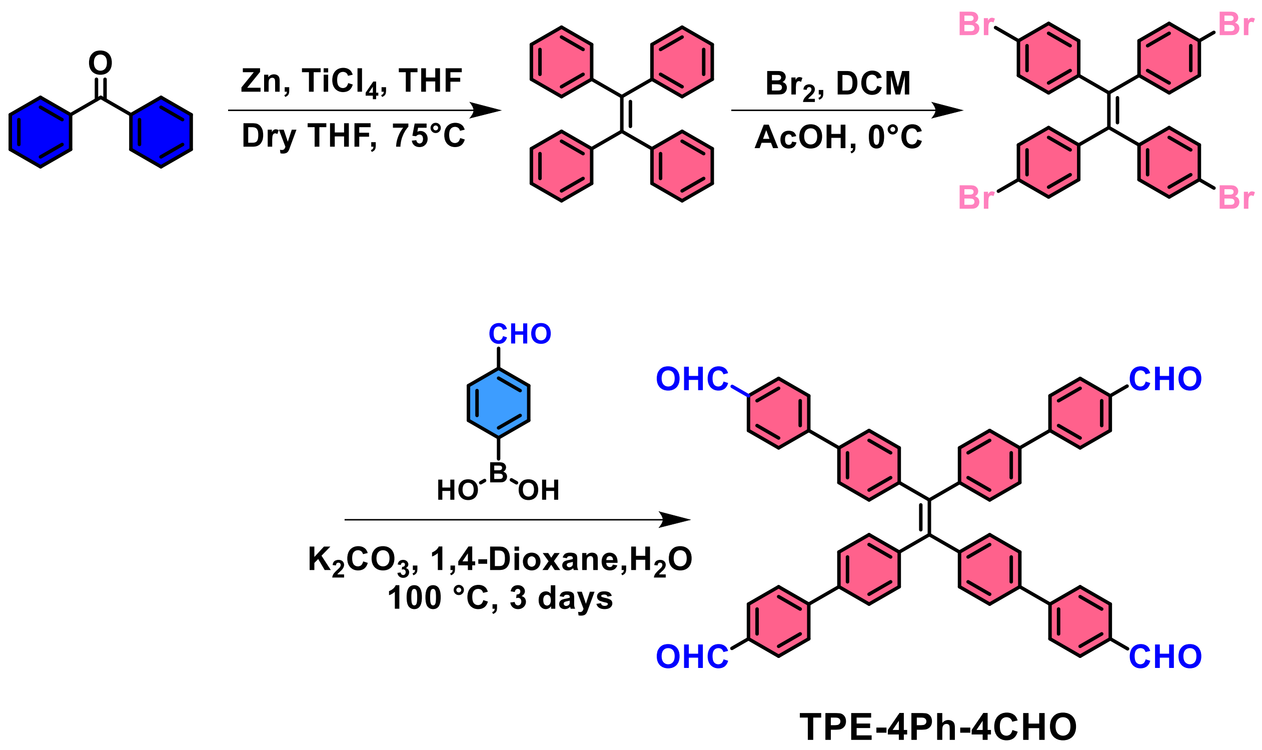

Scheme S1. Synthesis method of TPE-4Ph-4CHO.

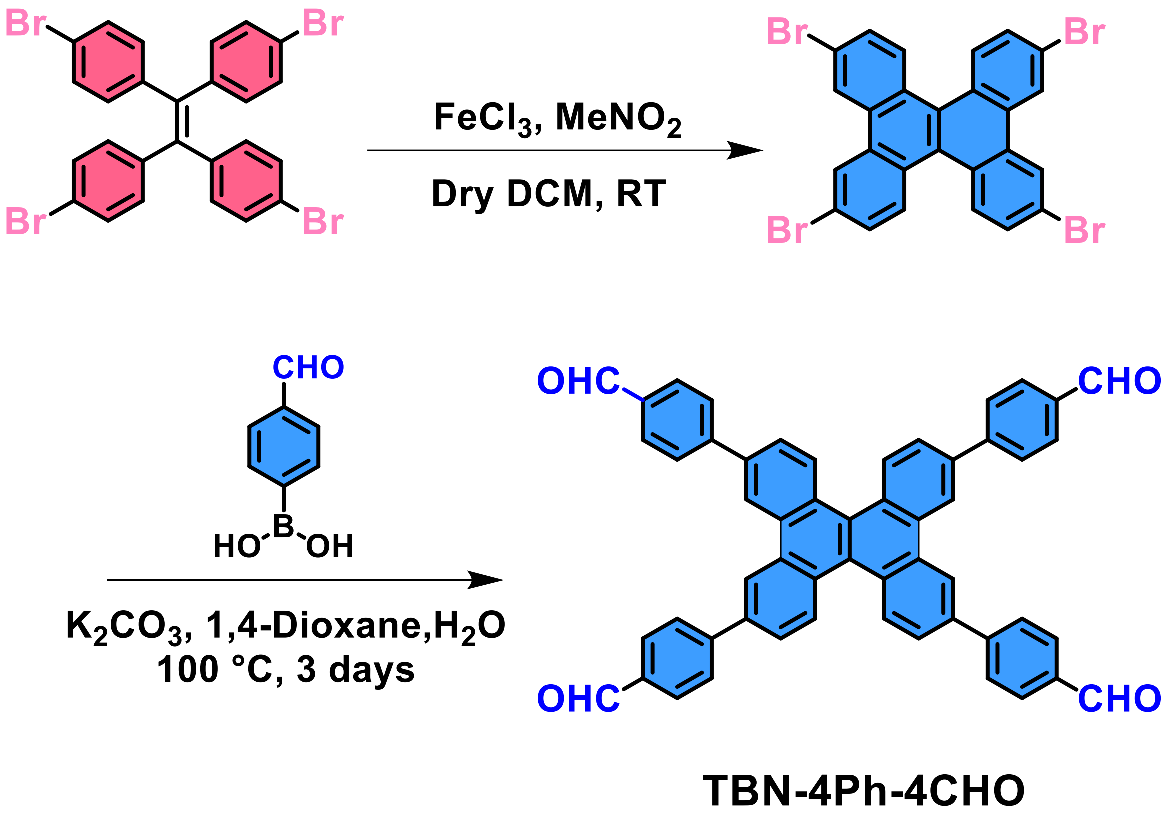

Scheme S2. Synthesis method of TBN-4Ph-4CHO.

Table S1. BET parameters of the as-synthesized TPE-TzTz CMP and TBN-TzTz CMP.

Samples	S_{BET} (m^2 g^{-1})	Pore size (nm)	Pore volume (cm^3 g^{-1})
TPE-TzTz CMP	484	1.11-1.8	1.2
TBN-TzTz CMP	419	1.46-2.69	0.2

Table S2. Values of $T_{d10\%}$ and Char yield of the as-synthesized TPE-TzTz CMP and TBN-TzTz CMP.

Samples	T_{d10} (°C)	Char Yield (%)
TPE-TzTz CMP	508	70
TBN-TzTz CMP	483	69

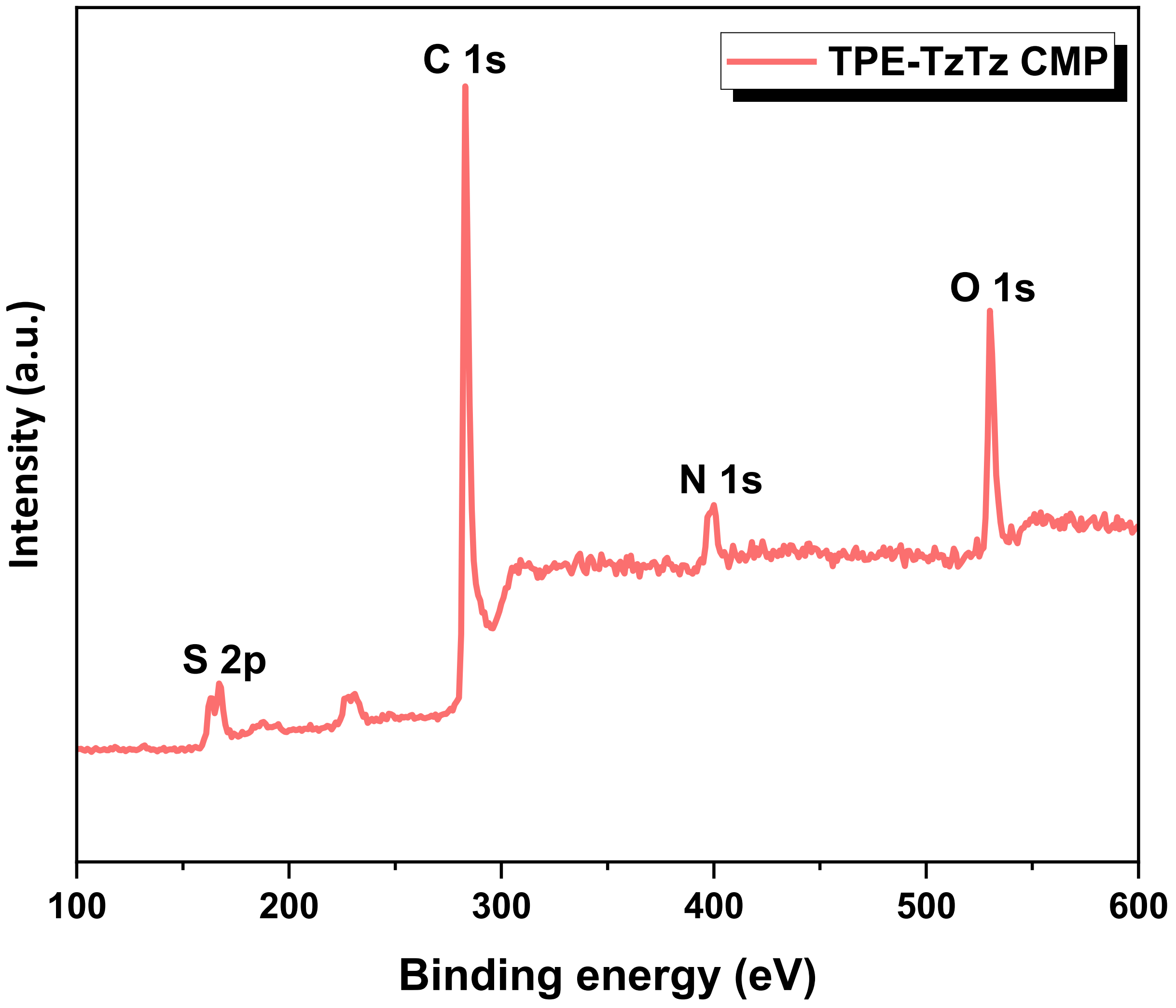

Figure S1. XPS survey spectrum of TPE-TzTz CMP.

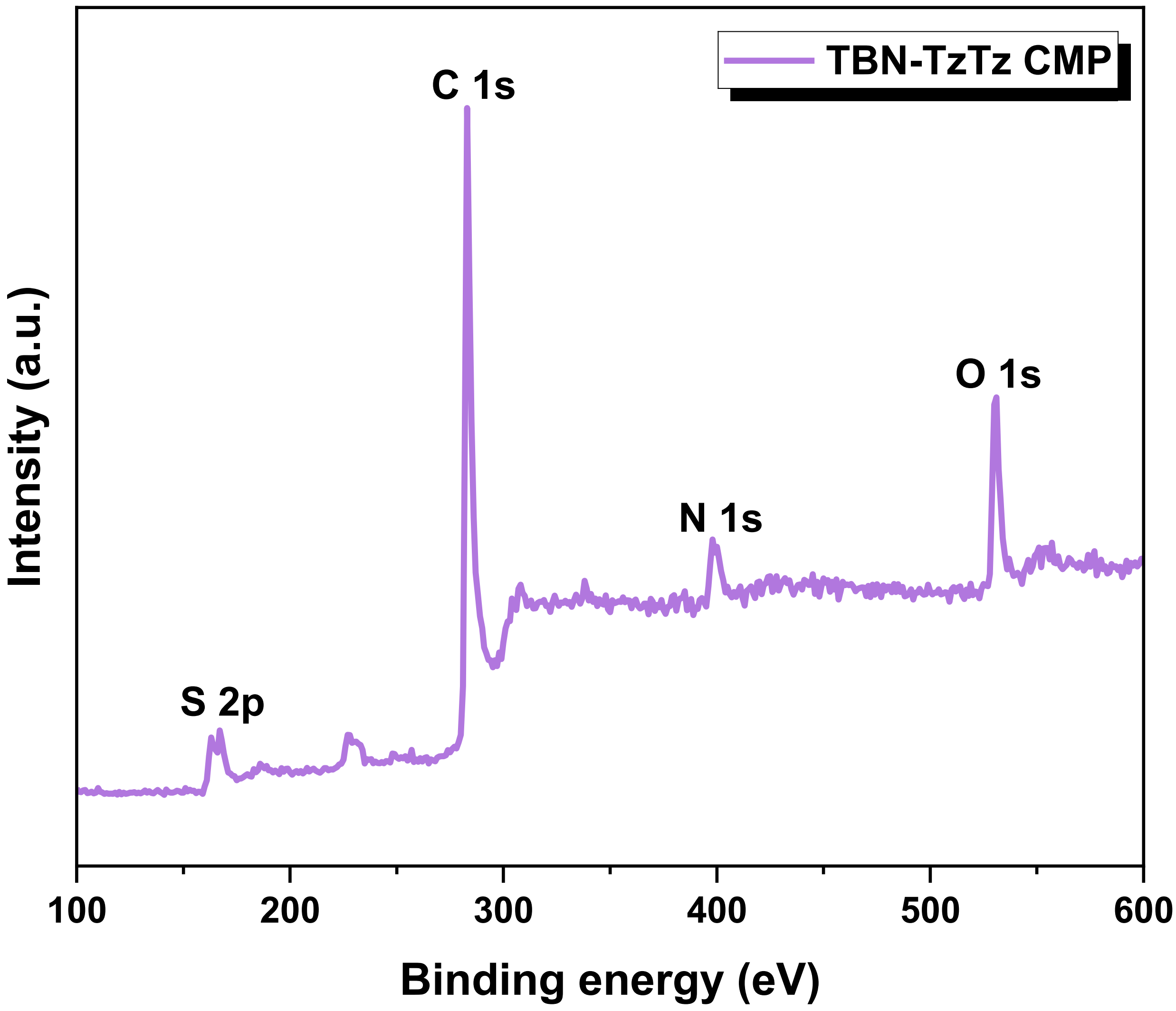

Figure S2. XPS survey spectrum of TBN-TzTz CMP.

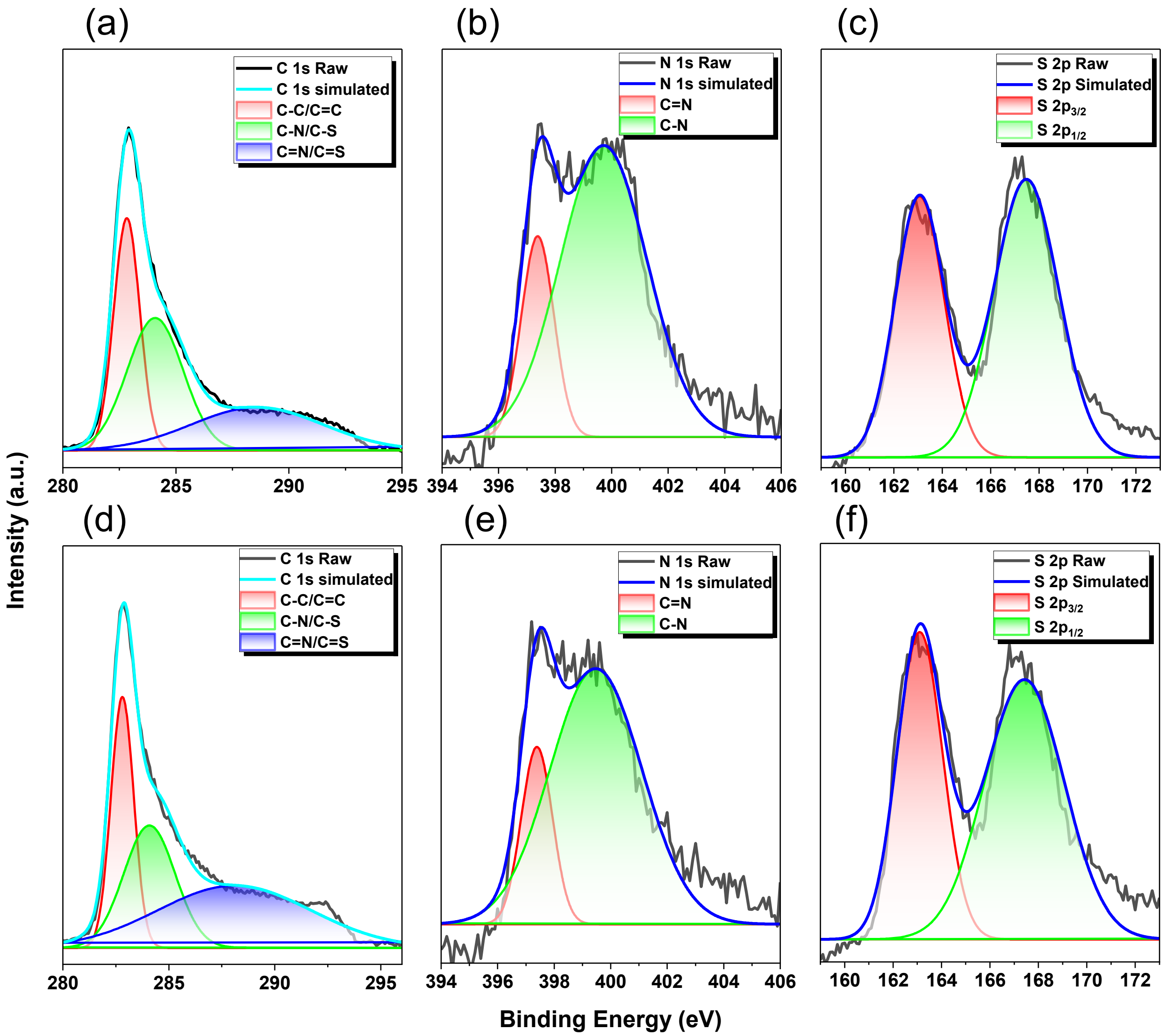

Figure S3. (a-f) high-resolution XPS spectra of C 1s, N 1s and S 2p for (a-c) TPE-TzTz CMP and (d-f) TBN-TzTz CMP.

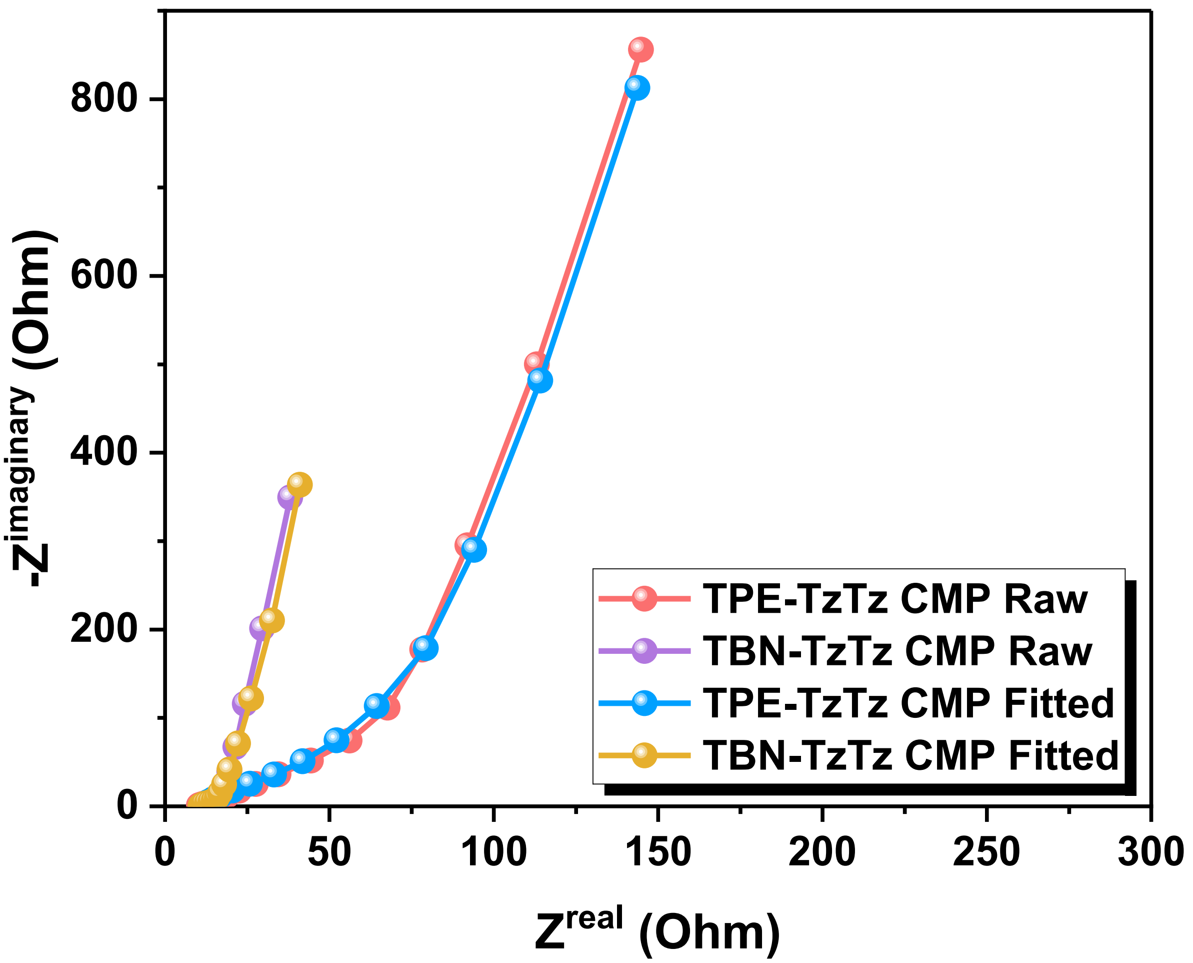

Figure S4. Electrochemical impedance spectroscopy (EIS) of TPE-TzTz CMP and TBN-TzTz-CMP.

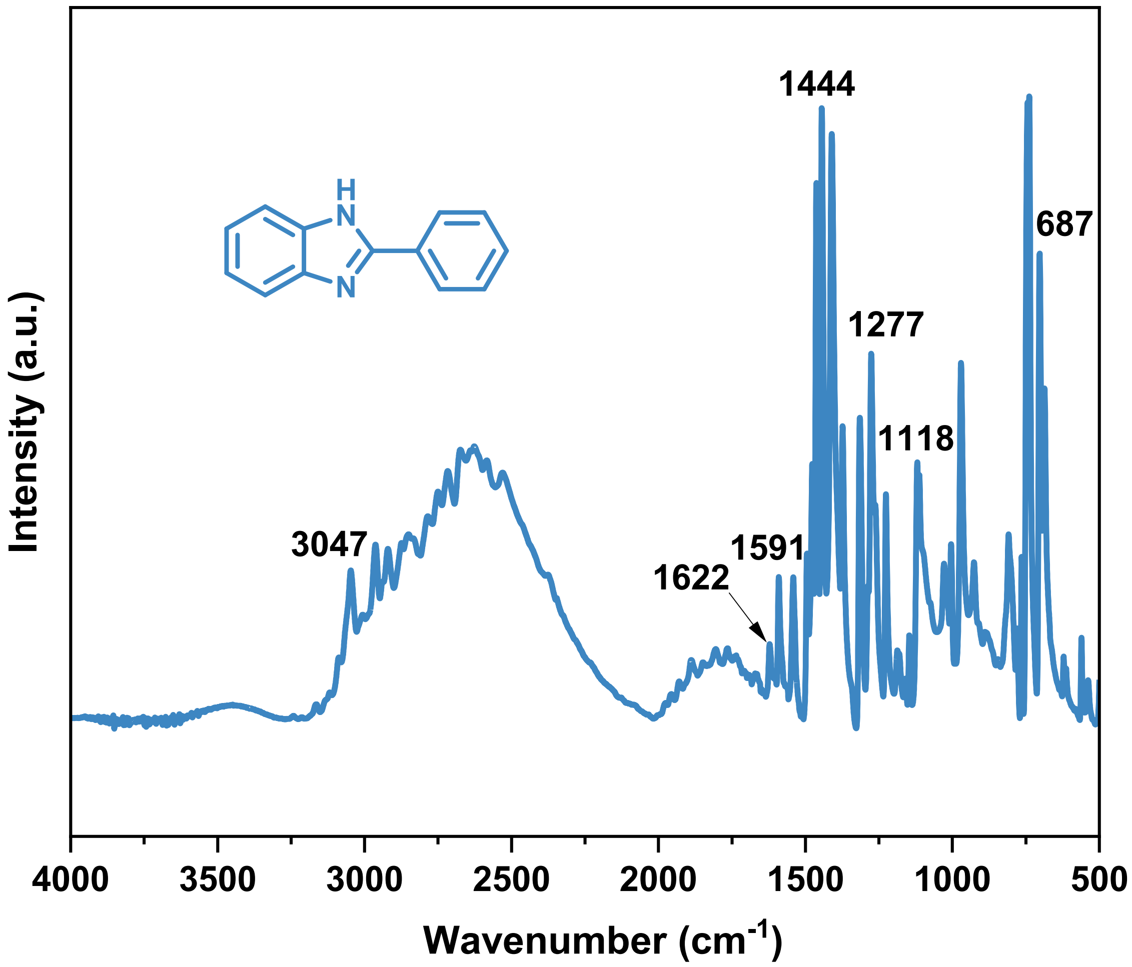

Figure S5. FTIR spectrum of 2-phenylbenzimidazole.

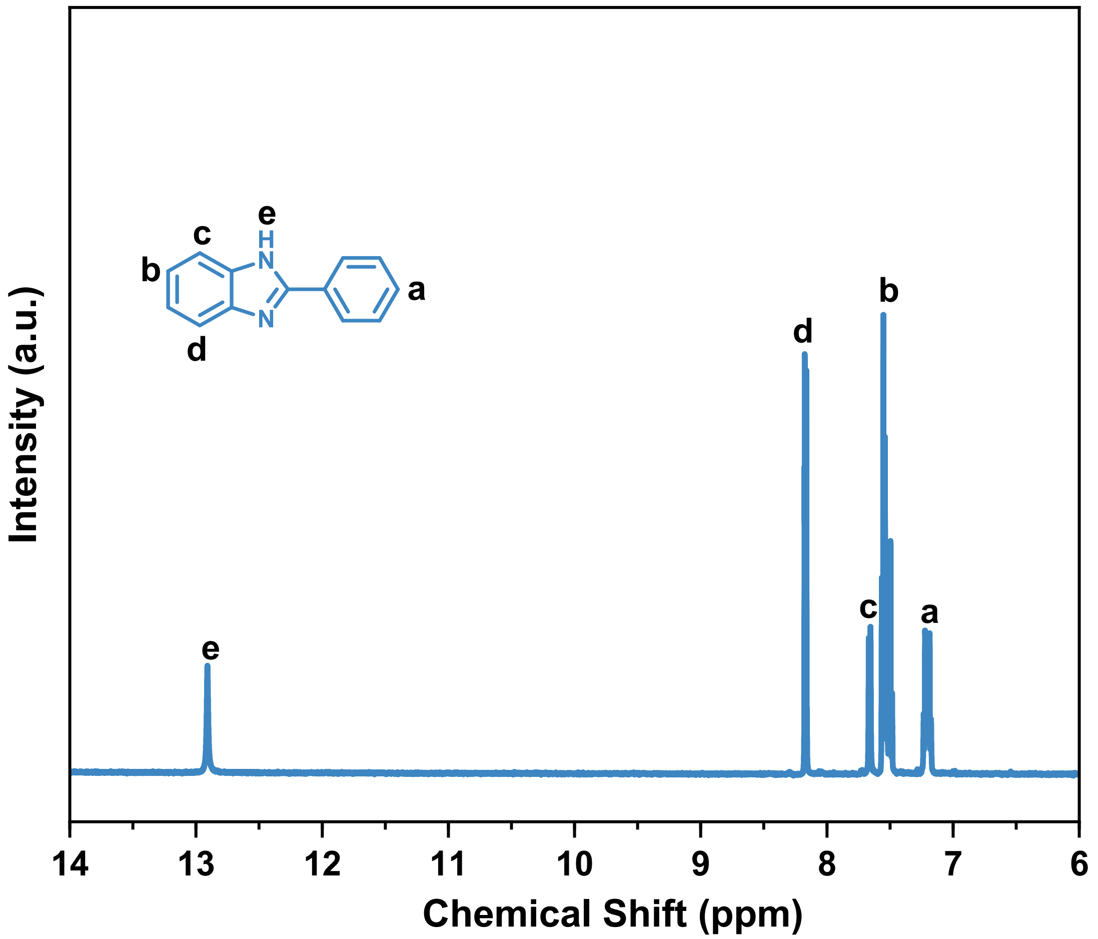

Figure S6. ^{1}H NMR spectrum of 2-phenylbenzimidazole.

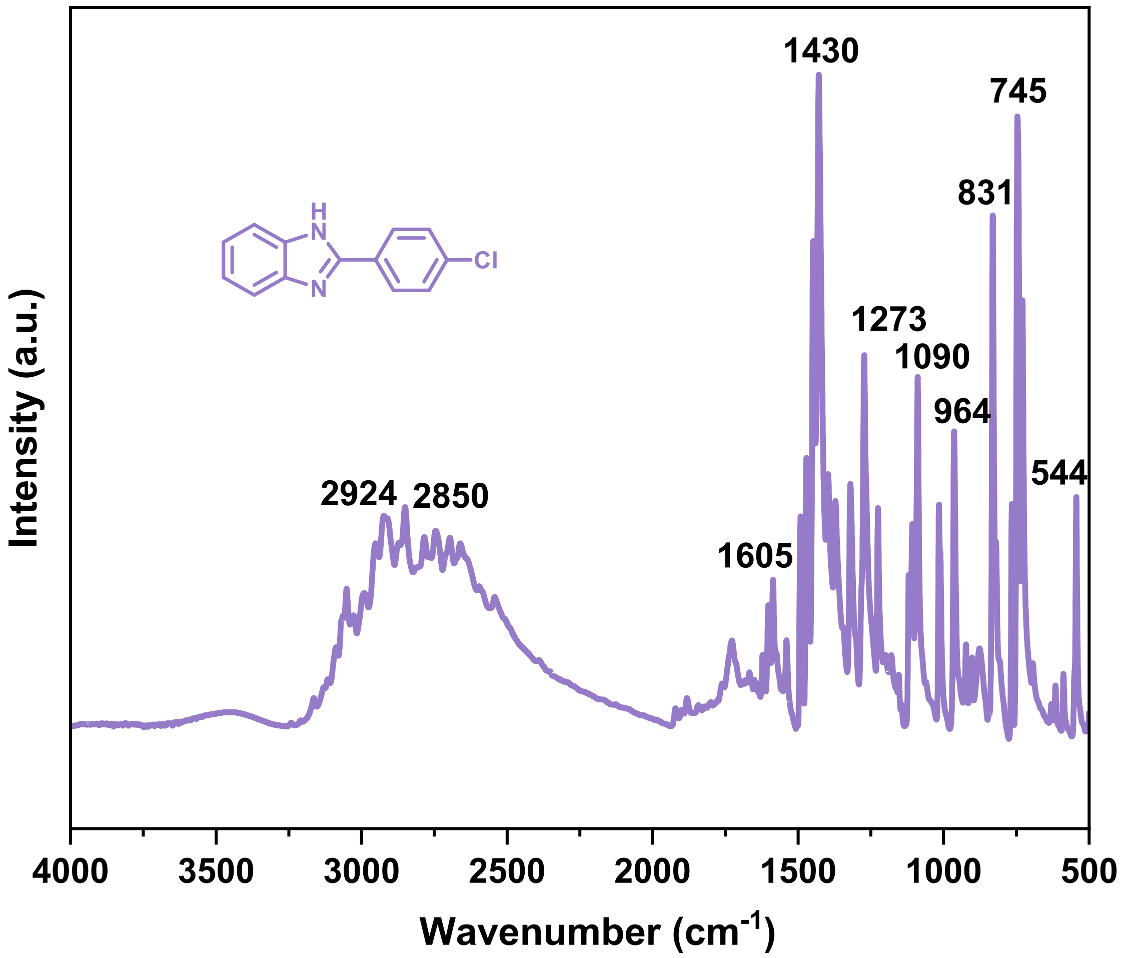

Figure S7. FTIR spectrum of 2-(4-chlorophenyl)benzimidazole.

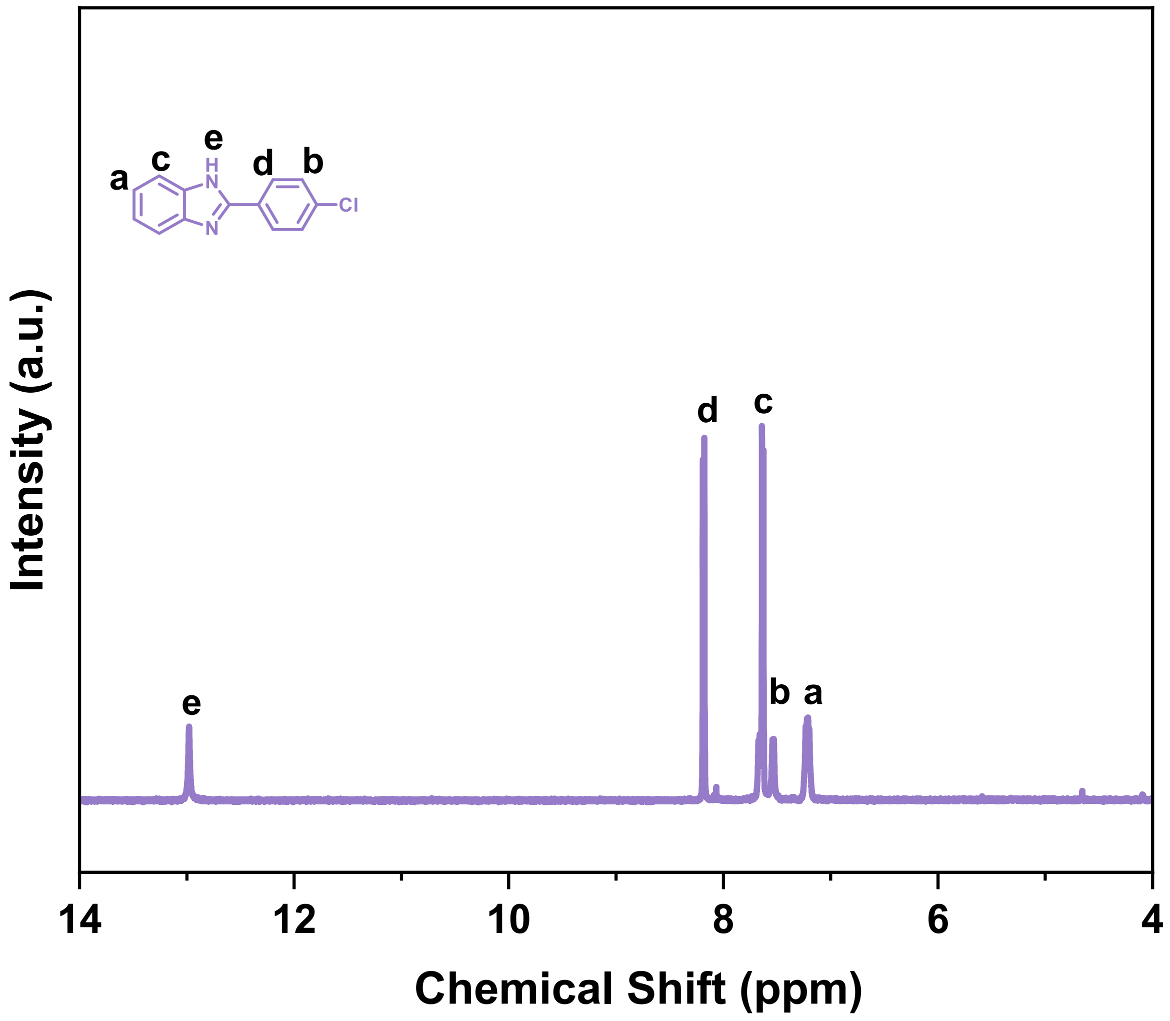

Figure S8. ^{1}H NMR spectrum of 2-(4-chlorophenyl)benzimidazole.

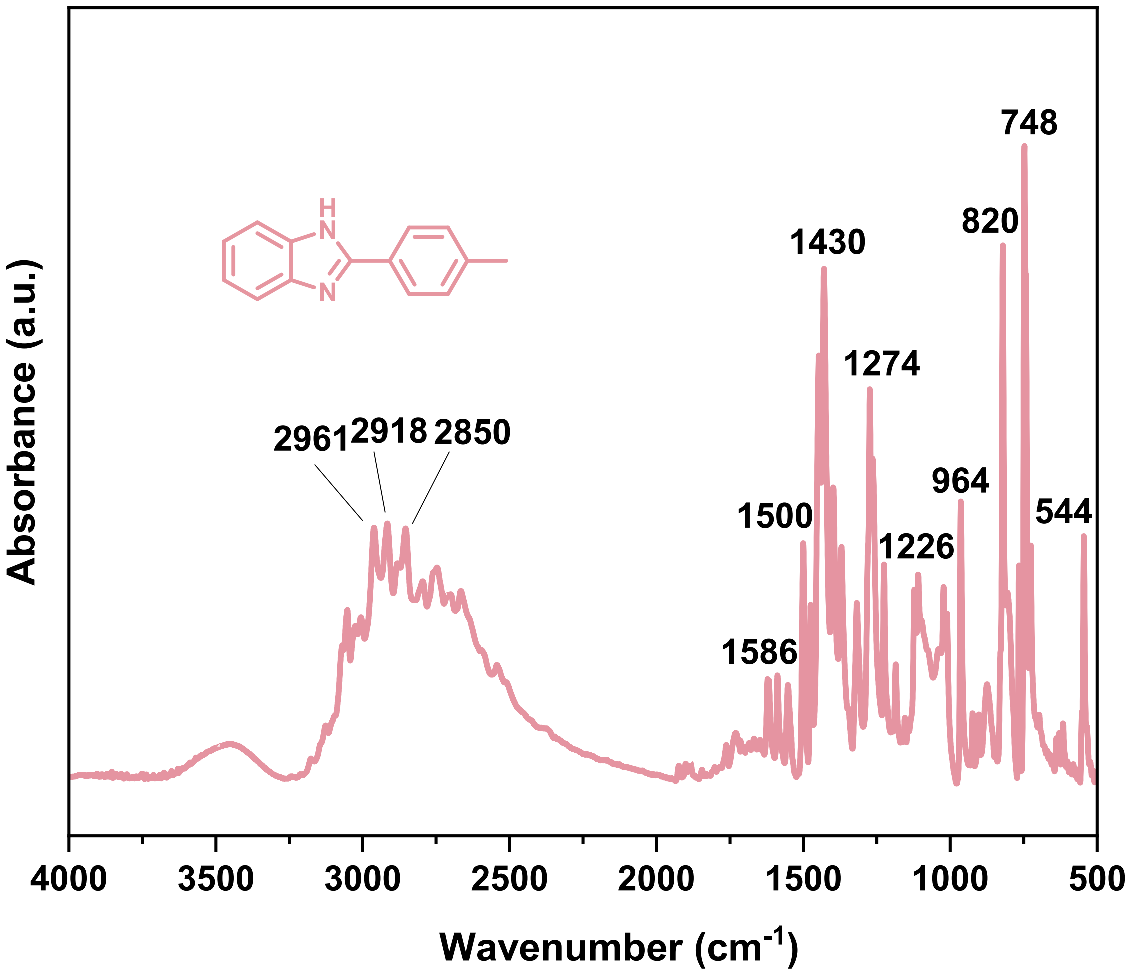

Figure S9. FTIR spectrum of 2-(4-tolyl)benzimidazole.

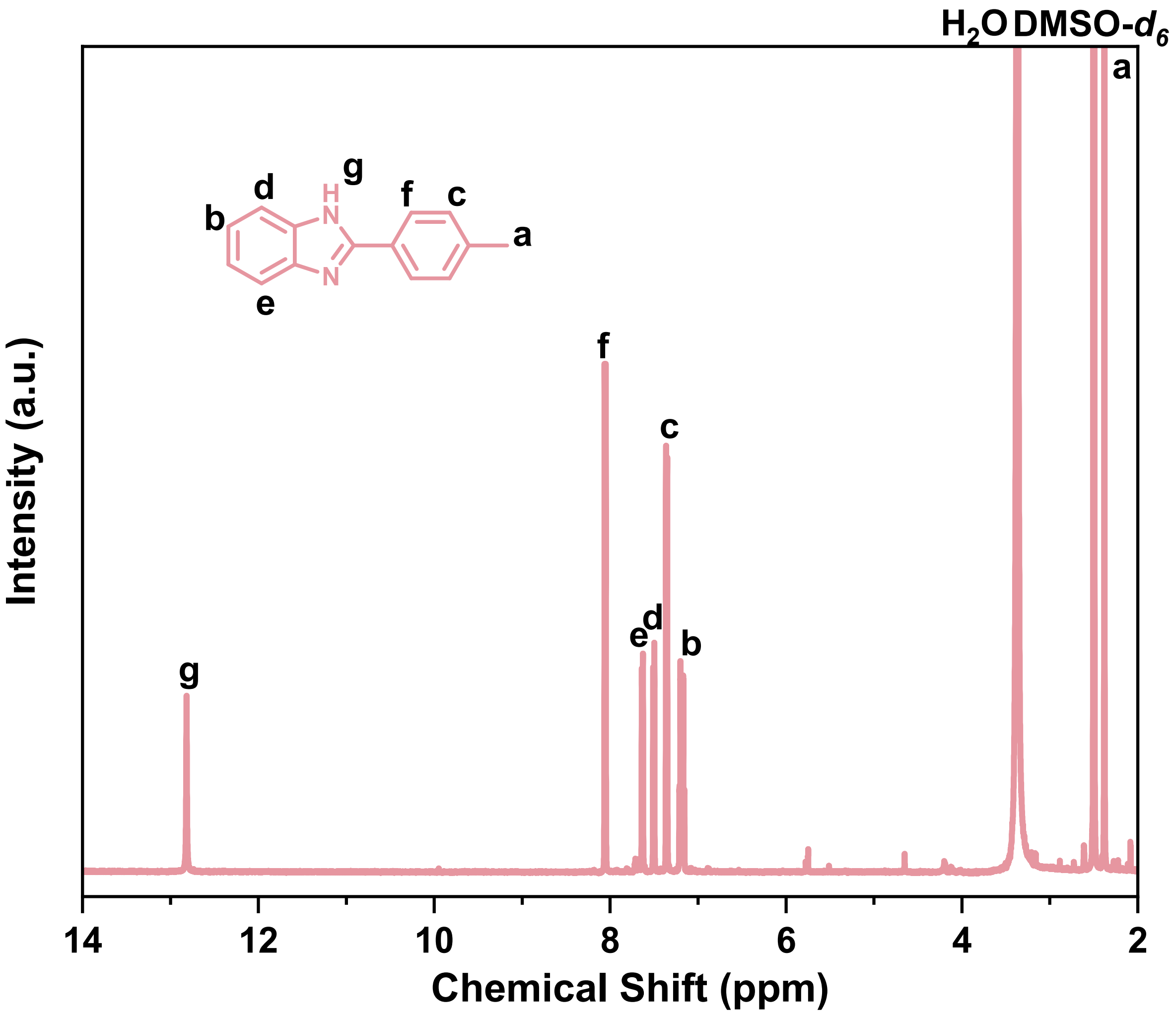

Figure S10. ^{1}H NMR spectrum of 2-(4-tolyl)benzimidazole.

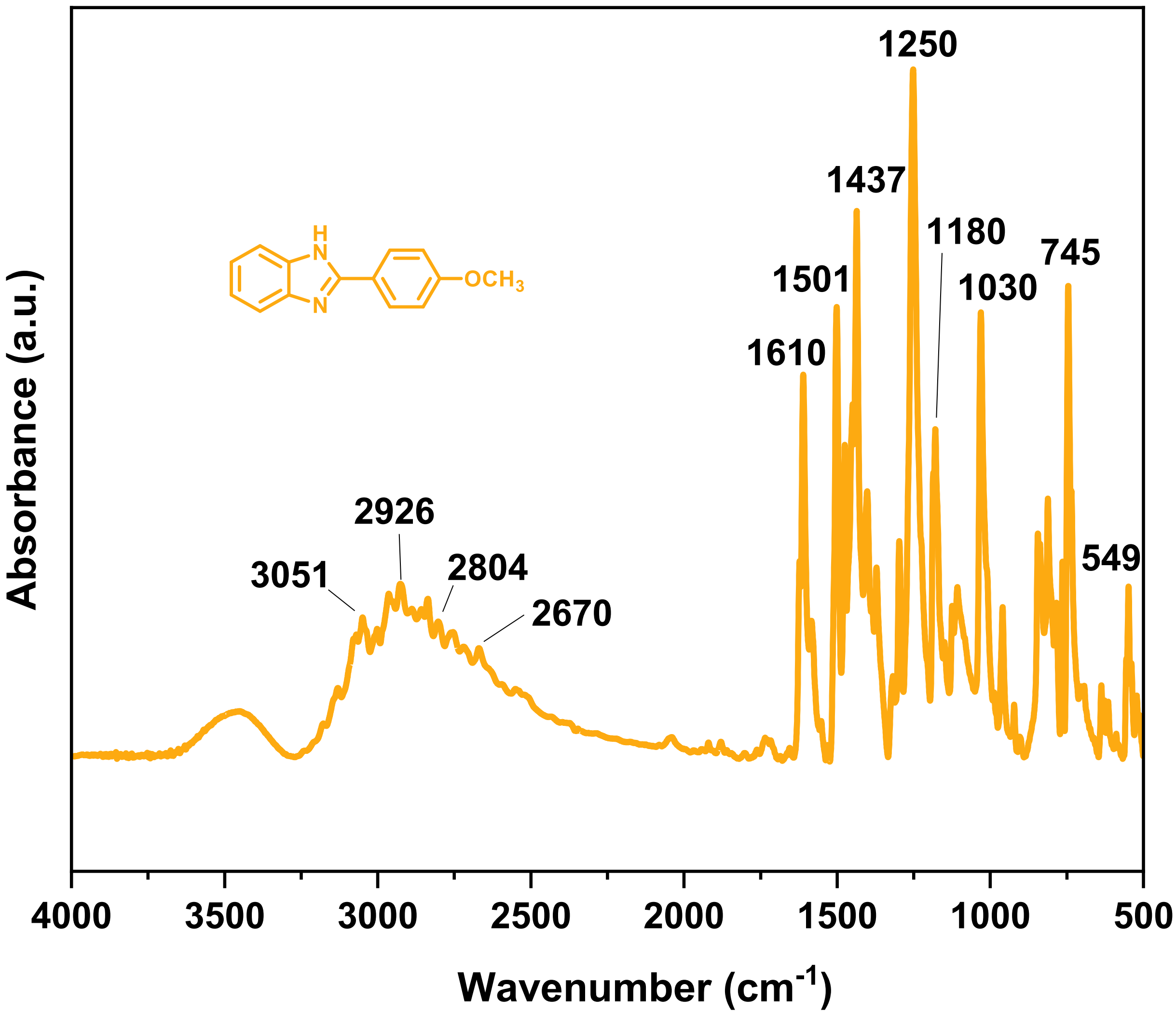

Figure S11. FTIR spectrum of 2-(4-methoxyphenyl)benzimidazole.

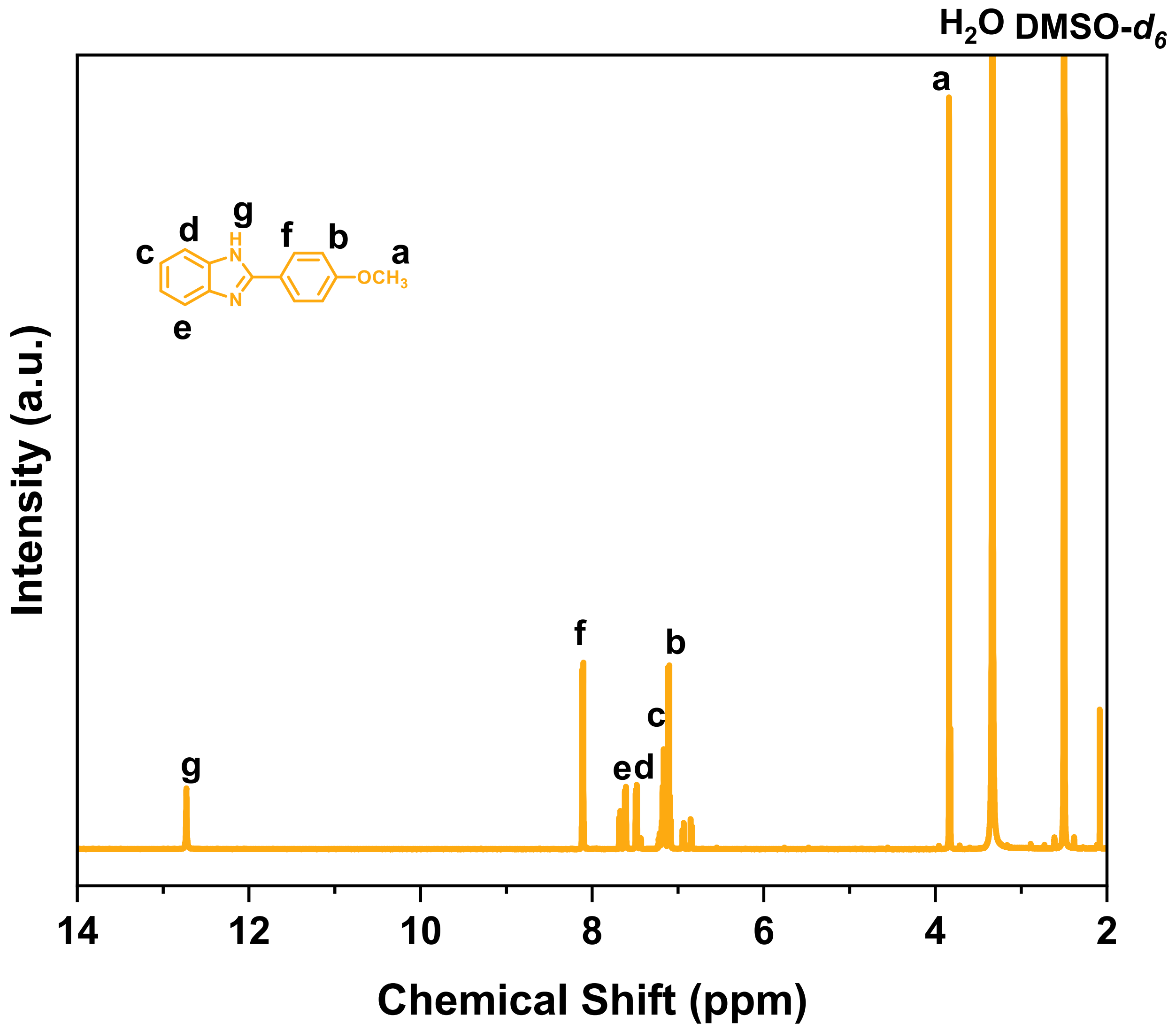

Figure S12. ^{1}H NMR spectrum of 2-(4-methoxyphenyl)benzimidazole.

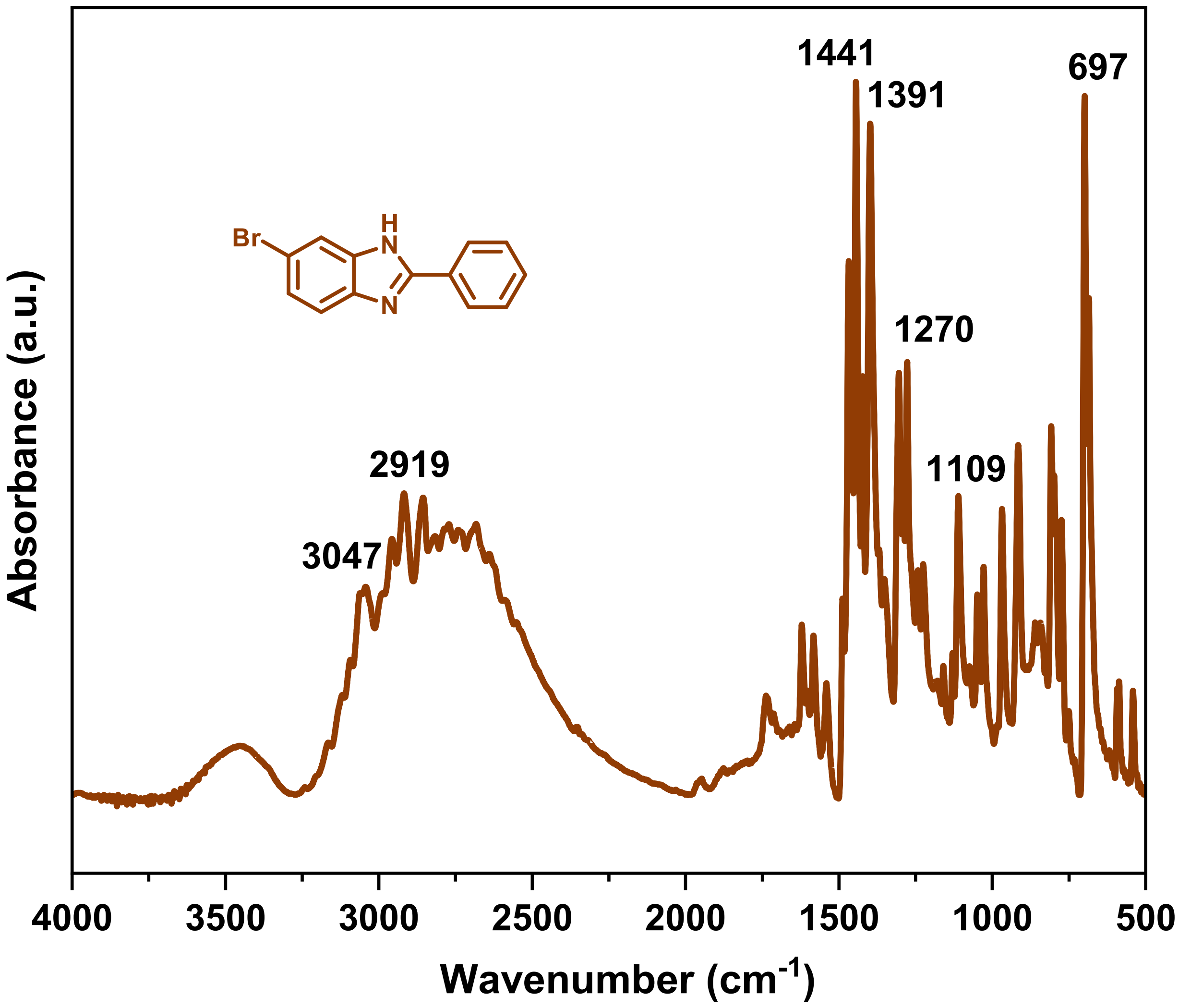

Figure S13. FTIR spectrum of 5-bromo-2-phenylbenzimidazole.

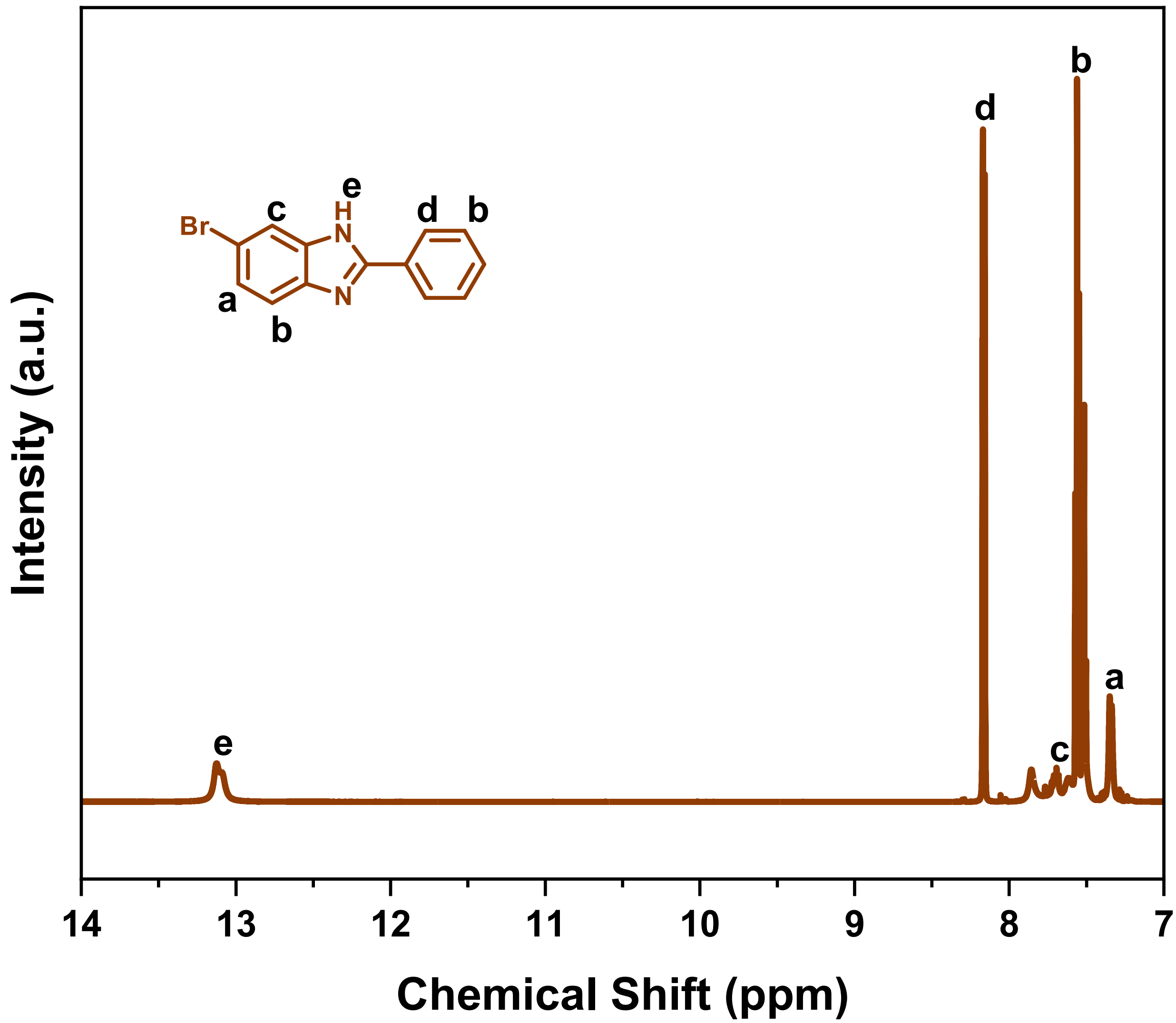

Figure S14. ^{1}H NMR spectrum of 5-bromo-2-phenylbenzimidazole.

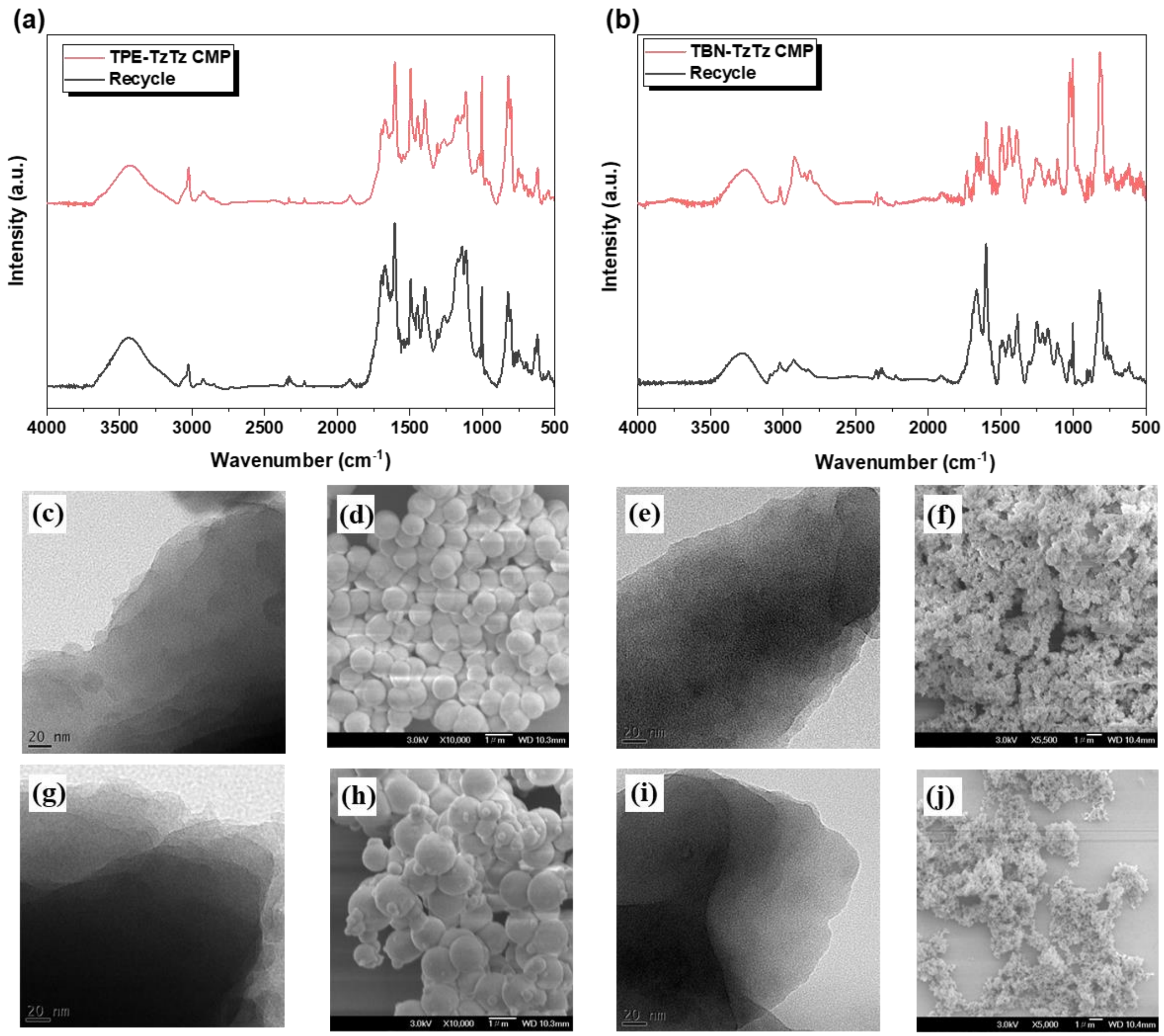

Figure S15. Comparison of FTIR spectra of (a) TPE-TzTz CMP and (b) TBN-TzTz CMP after the reaction cycles. TEM image of freshly prepared (c) TPE-TzTz CMP and (e) TBN-TzTz CMP, TEM image of the catalyst after reaction, TEM images of recycled (g) TPE-TzTz CMP (i) TBN-TzTz CMP, SEM image of freshly prepared (d) TPE-TzTz CMP and (f) TBN-TzTz CMP, and SEM image of the catalyst after reaction (h) TPE-TzTz CMP (j) TBN-TzTz CMP.

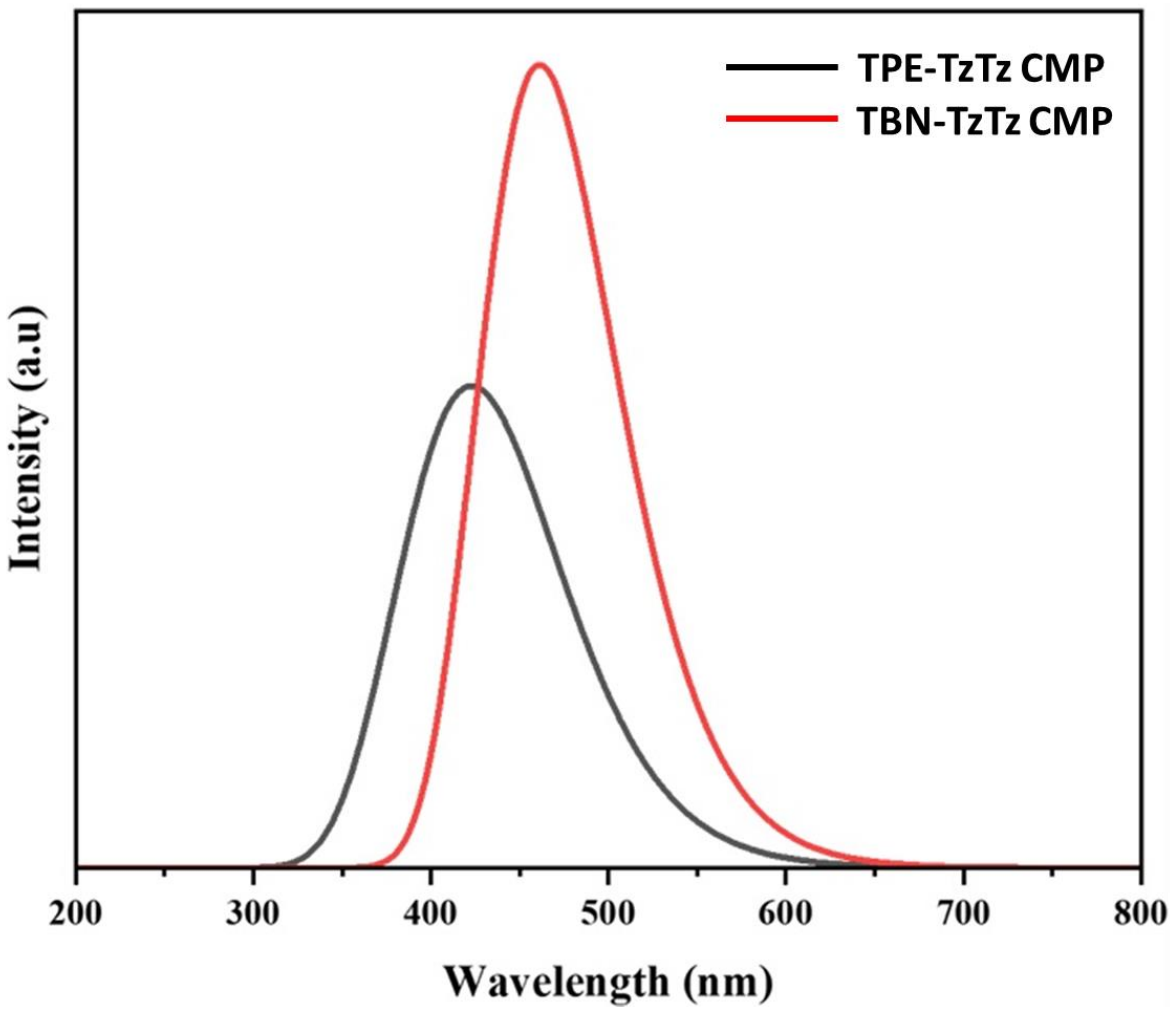

Figure S16. UV-Vis Spectra of TPE-TzTz and TBN-TzTz CMPs.

References

[1] Parr, R. G. Density Functional Theory. *Annu. Rev. Phys. Chem.* **1983**, *34*, 631–656. DOI: 10.1146/annurev.pc.34.100183.003215.

[2] Lee, C.; Yang, W.; Parr, R. G. Development of the Colle–Salvetti Correlation-Energy Formula into a Functional of the Electron Density. *Phys. Rev. B* **1988**, *37*, 785–789. DOI: 10.1103/PhysRevB.37.785.

[3] Karim, H.; Sardar, S.; Bibi, H.; Perveen, F.; Arfan, M.; Mumtaz, A. Effect of the Anionic Counterpart of Amino Acid Based Ionic Liquids upon Efficient CO_2 Capture: A Correlation of Experimental and DFT Study. *J. Mol. Liq.* **2024**, *405*, 125079. DOI: 10.1016/j.molliq.2024.125079.

[4] Frisch, M. J.; Trucks, G. W.; Schlegel, H. B.; Scuseria, G. E.; Robb, M. A.; Cheeseman, J. R.; Schlegel, H. B.; Scalmani, G.; Barone, V.; Mennucci, B.; Petersson, G. A. *Gaussian 09*, Revision C.01; Gaussian, Inc.: Wallingford, CT, **2010**.

[5] Lu, T.; Chen, F. Multiwfn: A Multifunctional Wavefunction Analyzer. *J. Comput. Chem.* **2012**, *33*, 580–592. DOI: 10.1002/jcc.22885.

[6] Humphrey, W.; Dalke, A.; Schulten, K. VMD: Visual Molecular Dynamics. *J. Mol. Graph.* **1996**, *14*, 33–38. DOI: 10.1016/0263-7855(96)00018-5.
